# Honokiol blocks tumor development and metastasis through mitochondrion-targeted effects

**DOI:** 10.1038/s41419-026-08441-6

**Published:** 2026-01-30

**Authors:** Martina Grandi, Francesco Boldrin, Giovanni Risato, Silvia Grillini, Natascia Tiso, Francesco Argenton, Emanuela Leonardi, Silvio Tosatto, Giancarlo Solaini, Alessandra Baracca, Valentina Giorgio

**Affiliations:** 1 Department of Biomedical and Neuromotor Sciences, University of Bologna, Bologna, I-40126 Italy; 2 Department of Biology, University of Padova, Padua, I-35121 Italy; 3 Department of Women’s and Children’s Health, University of Padova, Padua, I-35121 Italy; 4 Department of Biomedical Sciences, University of Padova, Padua, I-35121 Italy

**Keywords:** Molecular modelling, Apoptosis, Calcium channels

## Abstract

IF1 is the natural inhibitor of the mitochondrial ATP synthase during hydrolytic activity. It has been found to be overexpressed in many tumors, where it acts as a pro-oncogenic protein. During oxidative phosphorylation, IF1 binds to a novel site on the OSCP subunit of ATP synthase and promotes tumorigenesis by protecting cancer cells from permeability transition pore (PTP)-dependent apoptosis. In this work, honokiol, a biphenolic compound, showed binding affinity for two sites on the OSCP subunit, as predicted by molecular docking analysis. It was shown to be effective in disrupting the IF1-OSCP interaction and sensitizing cancer cells to apoptosis. In vivo, xenografts of zebrafish injected with IF1-expressing HeLa cells showed tumor development. The same xenografts, treated with honokiol, showed a significant reduction in tumor mass, similar to untreated fish injected with IF1 KO HeLa cells. In vitro, honokiol inhibits colony formation in soft agar of IF1-expressing HeLa cells by promoting the PTP opening and cell death, without any effect on cell proliferation. Interestingly, honokiol was shown to block metastasis in fish xenografts and migration in a wound healing assay, by promoting mitochondrial swelling in both control and IF1 KO cell lines, when cells are moving to close the scratch area. In conclusion, honokiol appears to be a promising anti-cancer compound, with pro-apoptotic properties through the displacement of IF1 from the OSCP subunit of ATP synthase, and anti-metastatic effects that are due to mitochondrial PTP opening.

## Introduction

Honokiol (HK), a plant bioactive compound, has been described for a broad range of anti-cancer activities in vitro and in vivo by regulating numerous signaling pathways. In vitro, HK was shown to inhibit the proliferation or viability of various cancer cells, including bone, brain, blood, colon, and lung cells, as reported in [1–8] and reviewed by [9]. It was shown to exhibit minimal cytotoxicity on normal cell lines, including human lymphocytes [10], fibroblast FB-1, FB-2, Hs68, and NIH-3T3 cells [11–14]. Furthermore, it is worth noting that HK can enhance the anti-neoplastic effects of several chemotherapeutic agents when cells are treated in combination [15, 16].

In in vivo studies, HK was proposed to inhibit tumor growth, metastasis, and angiogenesis in different animal models [9]. Some studies suggested that HK limits tumor-growth/proliferation by favoring apoptotic cell death [15, 17–22], but only a few of these studies proposed a plausible mechanism of its action [12, 23]. In breast cancer cells, early-stage apoptosis and programmed necrosis were shown to be activated by HK treatment through the expression of the receptor-interacting protein kinase 3 (RIP3) in parallel with an increase in Cyclophilin D (CyPD). The inhibition of CyPD by cyclosporin A (CsA) clearly blocked HK-triggered programmed necrosis. The effect of HK on the CyPD-regulated processes, but not on the expression of Bcl-xL and Bcl2 proteins [24], suggested that HK triggers programmed cell death through inner mitochondrial membrane permeability transition (PT). This is in line with a previous work showing that HK causes CyPD-dependent PT and apoptosis in HL-60, MCF-7 and HEK-293 cell lines [25]. In the human embryonic kidney cell line HEK‑293, HK was also shown to induce CyPD‑mediated necrosis associated with the inhibition of the mTOR signaling pathway [26]. More recently, HK was shown to enhance sirtuine 3 (SIRT3) expression and further triggers its activity. The decreased acetylation of mitochondrial SIRT3 substrates, superoxide dismutase (MnSOD) and the ATP synthase subunit OSCP, was paralleled by a beneficial increase in mitochondrial function in mouse hypertrophic hearts [27].

Cancer cell proliferation and migration largely rely on mitochondrial function and metabolic plasticity. Many cancer models inhibit cell death in response to fluctuations of physiological inducers of the mitochondrial PT pore (PTP), mainly Ca^2+^ and reactive oxygen species (ROS), or by controlling the PTP association with its physiological inducer, the CyPD [28–30], an ATP synthase modulator [31], which binds its OSCP subunit [28]. In vitro, the PT is accompanied by swelling of mitochondria, which was proposed to be mediated by the Ca^2+^-regulated pore in the 1970s [32, 33], and its basic regulatory features were defined in a series of seminal studies in 1979 [34–36]. More than 20 years later, ATP synthase complexes have been proposed to contribute to PTP formation [28, 37–42], although their involvement is still debated [43, 44]. Genetic manipulations of enzyme components have revealed modulatory mechanisms of the channel [45–51]. Moreover, we have recently shown that the natural ATP synthase inhibitor IF1, which is known to confer resistance to severe hypoxic conditions [52–54] in many tumors, can interact with the ATP synthase OSCP subunit and inhibit the PTP-dependent apoptosis [55] in respiring cancer cells.

In this study, we show that HK blocks tumor growth and metastases in vivo and in vitro by sensitizing PTP opening of cancer cells. The block of tumor growth through apoptosis appears to be mediated by the displacement of the inhibitor protein IF1 from its binding site on the OSCP subunit of ATP synthase, which is caused by the HK binding to the same OSCP region. On the other hand, the block of metastases was shown to be dependent on the activation of PTP opening through a mechanism that is independent of IF1.

## Results

### Honokiol binds to the OSCP subunit of ATP synthase and disrupts the IF1-OSCP interaction

HK was shown in different cancer cell models to promote CyPD-mediated cell death, but its binding site has not been defined so far. It modulates CyPD and SIRT3 activities and both these proteins interact with the OSCP subunit of ATP synthase [28, 51].

**Fig. 1:**
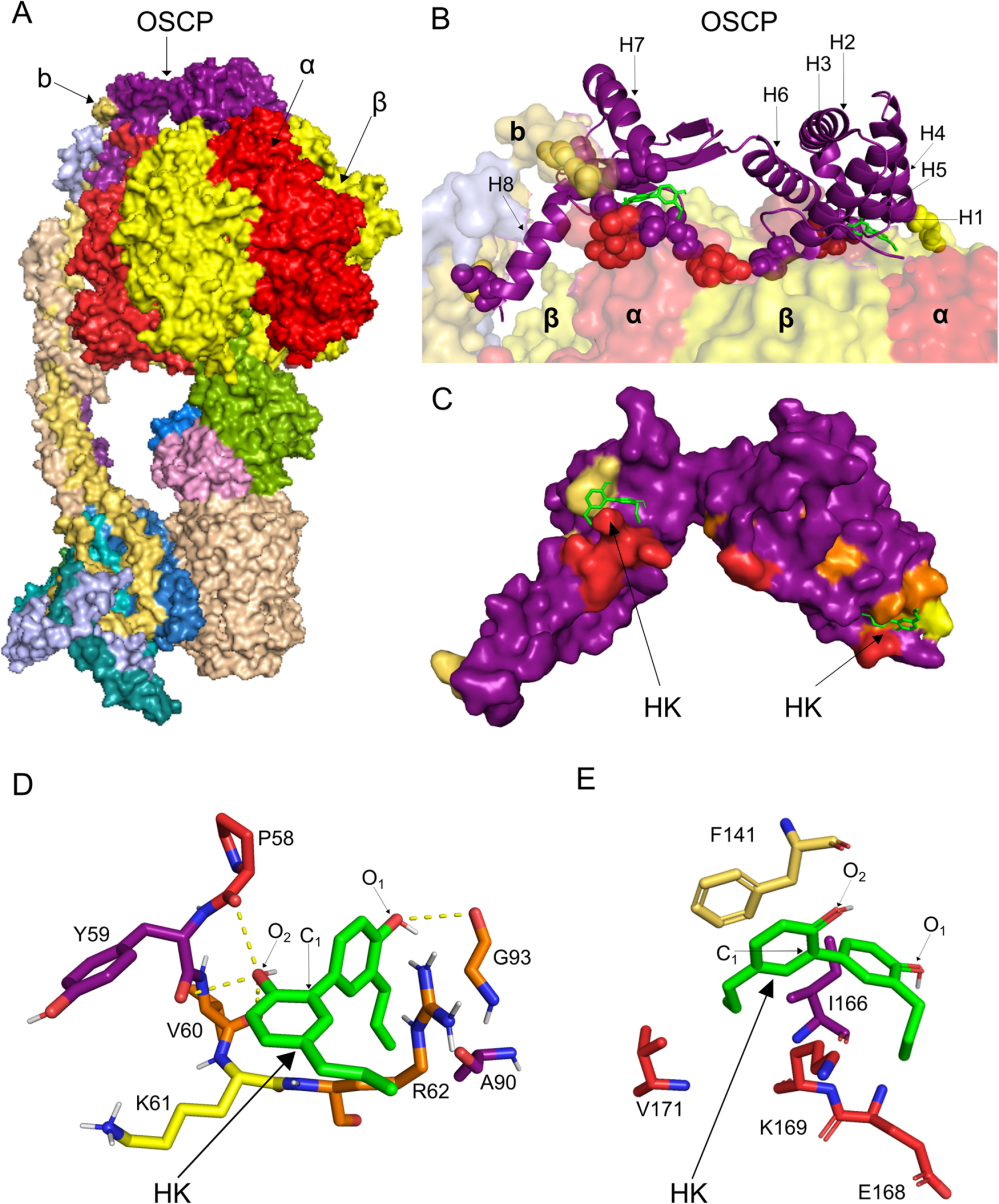
Honokiol is predicted to bind the OSCP subunit of ATP synthase. **A** Cryo-EM structure of the human ATP synthase complex (PDB ID: 8H9V) is presented during its catalytic state 3b (Lai Y. et al 2023). The subunits α, β, OSCP and b are red, yellow, purple and golden, respectively. **B** Three-dimensional (3D) structure of OSCP (chain O, residues 23-213) from the human ATP synthase complex is shown in purple. The OSCP α helixes 1-8 (H1-H8) are indicated. The aminoacidic residues of the OSCP subunit interacting with α, β, and b are in red, yellow, and golden, respectively. The two potential binding sites of HK (in green) with the highest binding affinity are shown, as predicted by AutoDock Vina 1.1.2. **C** The OSCP subunit structure is prepared in Chimera by removing other chains from the ATP synthase complex, non-standard amino acids, and water molecules. The residues are protonated by adding missing hydrogen atoms at pH 7.4. The OSCP residues interacting with IF1 or the ATP synthase subunits α, β, and b are in orange or in red, yellow, and golden, respectively. **D**, **E** The OSCP complexes with the two HK poses, at the N-terminal (**D**) or the C-terminal (**E**) regions of the subunit, are analyzed using RING v4.0 and used as input to build residue-interaction networks to detect non-covalent inter-chain interactions. HK is in green, its carbon and oxygen atoms (C_1_, O_1_, and O_2_) are labeled. The OSCP aminoacidic residues that are predicted to bind HK and are involved in interactions with other proteins are indicated in orange (IF1) or red, yellow, and golden (α, β, and b ATP synthase subunits), respectively. The OSCP residues in purple indicate none interaction with other proteins.

To test our hypothesis that HK might directly bind to the OSCP subunit, we performed in silico molecular docking simulations of HK on the OSCP protein structure. “Blind” docking calculations were performed using large box parameters on the human OSCP structure from the recently resolved cryo-EM structure of the human ATP synthase complex (PDB ID: 8H9V, chain O, residues 23–213). The OSCP subunit consists of an N-terminal α-helical domain and a C-terminal domain containing a β-sheet (Fig. 1A, B). The blind docking produced nine poses, organized into three clusters in the N-terminal domain and two in the C-terminal domain. The nine poses exhibited a predicted binding free energy ranging from −5.6 to −6.2 kcal/mol. The pose with the highest binding affinity (−6.2 kcal/mol) was located at a site formed by the extremities of α helices H3, H4, and H5 (Fig. 1B, C). The second-ranked pose, with a slightly lower binding affinity, was found in a pocket formed by the β-sheet and α helix H7 in the C-terminal domain (Fig. 1B, C).

A detailed analysis was then performed for the two binding sites to highlight key residues potentially involved in HK binding to the OSCP subunit. Analyses of residue interaction networks were conducted using strict and relaxed thresholds to identify key residues involved in HK binding in the two OSCP regions of interest. In the first site, seven residues at the N-terminus of the OSCP subunit (P58, Y59, V60, R62, K65, A90, G93) form van der Waals (VdW) contacts with HK (Fig. 1D and Supplementary Table 1). These contacts involve carbon, oxygen and hydrogen atoms of the HK interacting with the OSCP residue side chains (Supplementary Table 1). In the second site, HK forms VdW contacts with three residues (F141, K169, V171), involving only carbon atoms of HK and mainly hydrogen atoms of the OSCP residue side chains (Fig. 1E and Supplementary Table 1).

We therefore investigated the role of the OSCP residues involved in the HK binding on the ATP synthase structure by mapping the OSCP regions in contact with other ATP synthase subunits or with the inhibitor IF1 (Fig. 1B, C). This analysis revealed that the two poses with the highest binding affinity are located in areas of the OSCP that interact with other ATP synthase subunits [56]. In the first pose (Fig. 1D), HK contacts the P58 residue on the OSCP N-terminal domain, which forms a VdW interaction with the ATP synthase α subunit. However, in this pose, HK forms contacts with the three exposed residues, V60, G93, and R62, which have been experimentally determined to bind IF1 with low affinity during oxidative phosphorylation (Fig. 1C, D) [55]. This finding suggests that HK might compete with IF1 for its binding to the OSCP subunit.

In the second pose (Fig. 1E), HK is positioned within a pocket between the β-sheet and the helix H7 on the OSCP C-terminal domain. Here, HK makes VdW contacts with residues K169 and V171 of the β-sheet that interact with the ATP synthase α subunit, and with residue F141 of H7, which contacts the ATP synthase b subunit (Fig. 1B, E). It is important to note that this analysis has been performed using the ATP synthase structure during its catalytic activity (state 3b [57]) and pH 7.4. The contacts occurring between OSCP and other ATP synthase subunits change through the catalytic states of rotation (Supplementary Table 2). The analysis of the residue interaction networks performed in the different states of ATP synthase, highlighted that OSCP contacts both α and β subunits of the F1 head domain and subunits of the peripheral stalk (subunit b), with some states presenting more contacts with β subunits and the peripheral stalk components, and others showing higher residue availability for the IF1 or HK binding to the OSCP subunit (Supplementary Table 2, state 3a, b). Since the available ATP synthase structure was determined under ATP hydrolysis condition, we assume that under ATP synthesis condition, the conformation of OSCP remains stable, being a component of the stator of the enzyme complex.

**Fig. 2:**
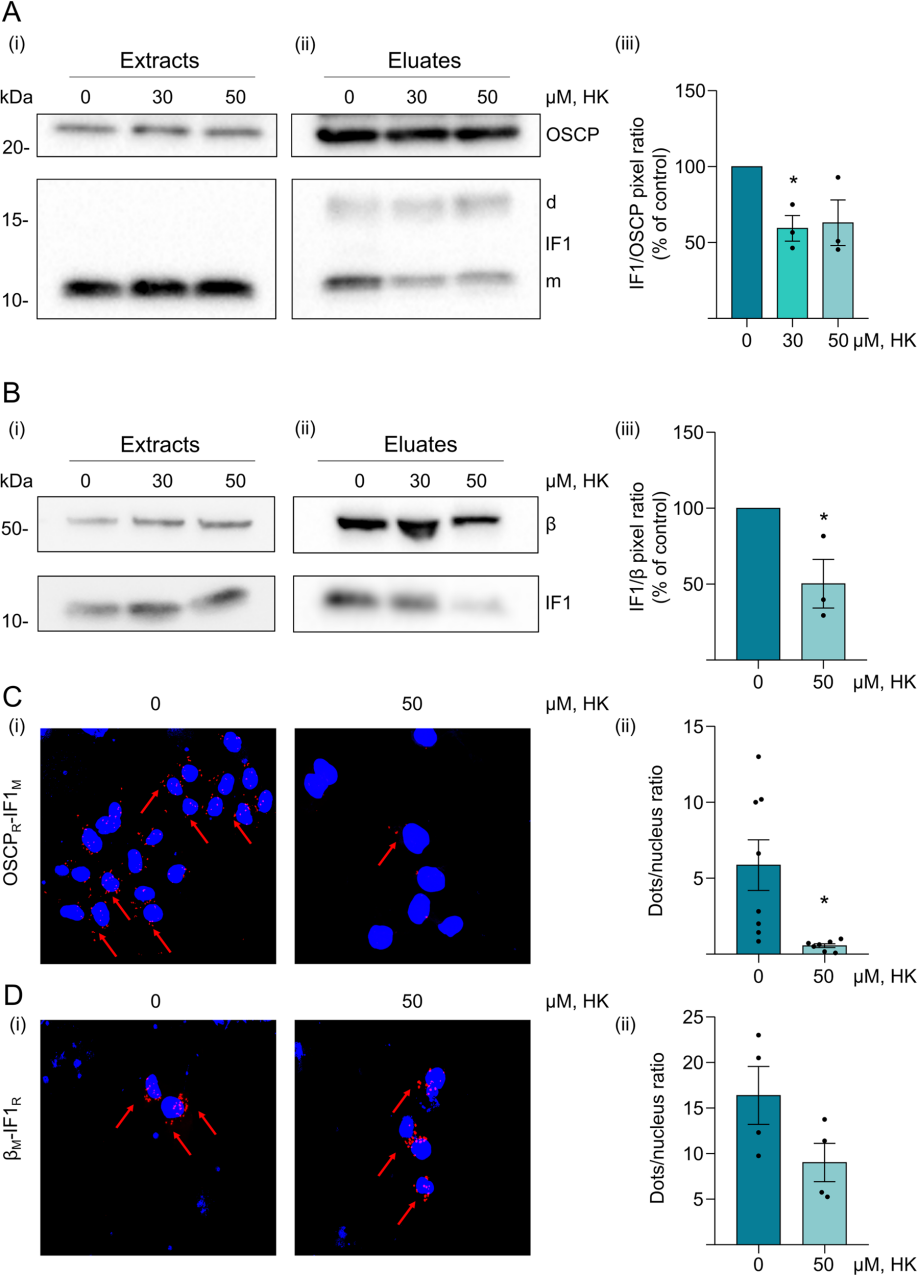
Honokiol displaces IF1 from ATP synthase. **A** Permeabilized wild-type HeLa cells are incubated in an ADP-regenerating buffer in the presence of 0–50 μM HK and are immunoprecipitated for the OSCP subunit. Western blotting shows the OSCP subunit and IF1 contained in the mitochondrial extracts (Extracts, (i)) and in the immunoprecipitated fractions (Eluates, (ii)). For IF1 monomeric or dimeric forms, m or d are indicated, respectively. Histogram (iii) shows the mean of IF1/OSCP pixel ratio (expressed as % of control) in the eluates. Data are mean ± SEM of three independent experiments. P value is *p = 0.0479, One-way Anova. **B** Mitochondria isolated from wild-type HeLa cells are incubated in an ADP-regenerating buffer in the presence of 0-50 μM HK and are solubilized in a 1% (w/v) digitonin-containing buffer. Mitochondrial extracts are subjected to immunoprecipitation of ATP synthase. Western blotting shows the β subunit and IF1 contained in the digitonin mitochondrial extracts (Extracts, (i)) and in the immunoprecipitated fractions (Eluates, (ii)). Histogram (iii) shows the mean of IF1/β pixel ratio (expressed as % of control) in the eluates. Data are mean ± SEM of three independent experiments. P value is *p = 0.0359, Student’s *t* test. **C**, **D** Representative images of CTR HeLa cells untreated or treated with 50 µM HK are analyzed by Proximity Ligation Assay (PLA) to assess protein-protein interactions (i). The antibody combinations applied to test the interactions between mitochondrial proteins are indicated on the left. They detect OSCP and IF1 (**C**, (i)) or β subunit and IF1 (**D**, (i)) interactions. M or R indicate the secondary antibodies used during the PLA protocol. Interactions are revealed by red dots, while DAPI-stained nuclei are in blue. PLA analysis quantification (ii) shows the number of red fluorescent dots per nucleus in CTR HeLa cells. Data are mean ± SEM of three independent experiments. *p = 0.0113, Student’s *t* test.

To investigate the occurrence of HK interactions with the OSCP subunit in cancer cells, and its possible effects on the IF1 binding to this subunit, mitochondria derived from wild-type HeLa cells were treated with increasing concentrations of HK (Fig. 2A, B).

HK treatments were defined by titration for the different experimental conditions as detailed in Materials and Methods section. Freshly prepared mitochondria were incubated in an ADP-regenerating buffer (State 3 steady state respiratory condition, Fig. 2A, B) to promote the IF1-OSCP subunit interaction [55], and their extracts were immunoprecipitated for either the OSCP subunit (Fig. 2A) or the F1 catalytic domain of ATP synthase (Fig. 2B). Monomeric IF1 immunoprecipitated (eluates) with the OSCP subunit when both the OSCP- or the F1 domain- targeted antibodies were used for immunoprecipitation (Fig. 2A, B, (ii)). A band corresponding to IF1 dimer was detected through Western blotting only in the OSCP (Fig. 2A (ii)), due to different detergent and salt conditions. Increasing concentrations of HK (Fig. 2A, B (ii)), that were used to treat mitochondria under active oxidative phosphorylation (for 10 min), caused an IF1 displacement from the OSCP subunit (Fig. 2A (ii)) and from the whole ATP synthase complex (Fig. 2B (ii)), which included the OSCP subunit [28].

The effect of HK in displacing the IF1-OSCP interaction was further tested in situ by proximity ligation assay (PLA) (Fig. 2C). Adherent HeLa cells, treated with 50 µM HK for 30 min before cell fixing, showed a decrease in IF1-OSCP interaction, as revealed by red fluorescent dots and their quantification (Fig. 2C (i-ii)). On the contrary, HK treatment in situ did not significantly affect the IF1-β subunit interaction (Fig. 2D (i-ii)), indicating that the displacing action of HK is selective for the IF1-OSCP interaction.

These findings strongly indicated that HK causes the IF1 release from its binding site on the OSCP subunit, previously characterized in HeLa cells [55]. Moreover, the results showed that the prediction of the HK binding to the OSCP residues V60, R62, and G93 (Fig. 1D) is plausible in treated HeLa mitochondria, where HK disrupts the IF1-OSCP interaction, which falls within the same V60, R62, and G93 residues of the N-terminus of the OSCP subunit, as characterized by NMR analysis [55].

### Honokiol treatment prevents tumor growth and metastasis in vivo

**Fig. 3:**
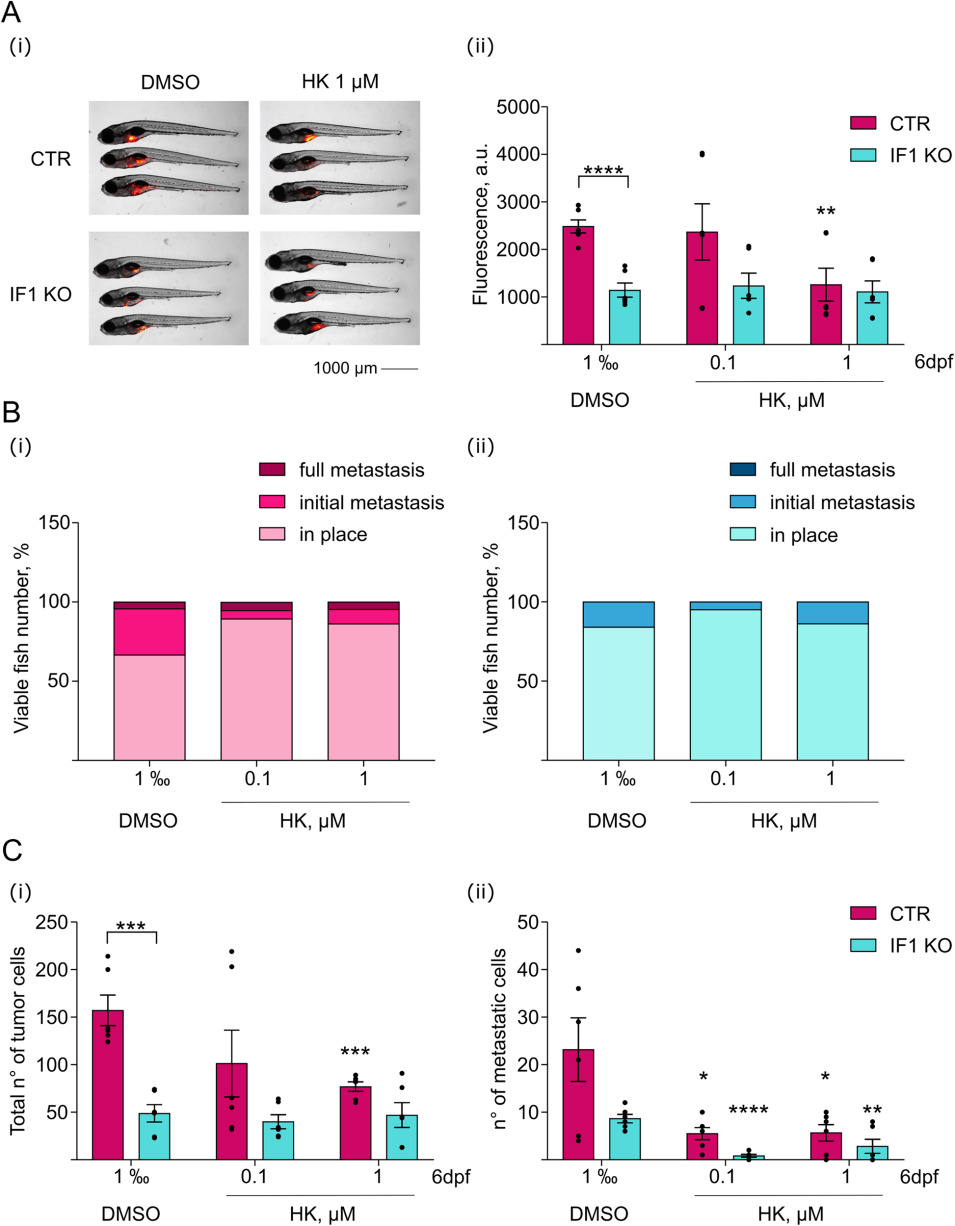
Honokiol causes a decrease of tumor mass and metastases in vivo. **A** CTR and IF1 KO HeLa cells, are injected into the yolk of zebrafish aged 2 days post fertilization (dpf). Fish are treated with 1‰ DMSO, 0.1 or 1 µM HK. Images (i) of fish (DMSO and 1 µM HK) at 6 dpf are shown. Red fluorescence indicates tumor masses derived from HeLa cells. Scale bar, 1 000 μm. Histogram (ii) represents the mean of fluorescence at 6 dpf of HeLa cells. Fluorescence indicates tumor mass development in fish treated with 1‰ DMSO, 0.1 or 1 µM HK. Data are mean ± SEM of six independent experiments. **p = 0.0081, ****p ≤ 0.0001, Student’s *t* test. **B** Histograms represent the % of fish injected with CTR (i) and IF1 KO (ii) HeLa cells showing tumor in place, or initial and full metastasis. **C** Total number of CTR or IF1 KO HeLa cells forming primary tumor (i) or metastases (ii) in zebrafish (6 dpf) injected as above. Data are mean ± SEM of six independent experiments, *p ≤ 0.05, **p = 0.0072, ***p ≤ 0.001, ****p ≤ 0.0001, Student’s *t* test.

The IF1 inhibitor favors tumor formation and growth in many different cancer models. Since HK caused the IF1 displacement from the OSCP subunit (Fig. 2A), and this might affect the cell apoptosis and cancer progression [55], the effects of HK treatment were studied in zebrafish xenografts (Fig. 3A). In vivo models were generated by injection of control or IF1 KO HeLa cells in zebrafish embryos at 2 days post fertilization (dpf). We took advantage of the IF1 KO HeLa cell population and its respective control, obtained by a CRISPR-associated protein 9 -based genetic approach. IF1 KO HeLa model was previously characterized and did not show any difference in the amount of ATP synthase subunits, nor in the tested mitochondrial membrane or matrix proteins, compared to controls [55], with the only variance being the lack of IF1.

HeLa cell fluorescence, which is an index of tumor growth in zebrafish xenografts, was measured upon injection (2 dpf), and 4 days after it (6 dpf). Fish were grown in fish water in the presence of treatment during tumor growth. The tumor mass significantly decreased upon treatment with 1 µM HK of fish injected with control HeLa cells, but not in fish injected with IF1 KO cells, which did not develop large tumor masses, as the controls did (Fig. 3A (i), (ii)). The fish number with metastases was reduced by both the lack of the inhibitor protein IF1 or by HK treatment in the control HeLa-injected embryos (Fig. 3B (i), (ii)). The IF1 KO xenografts, developing metastases, were slightly decreased in number by HK treatment (Fig. 3B (ii)). Moreover, HK treatment reduced the total number of the control HeLa cells (Fig. 3C (i)) forming tumor masses, but not that of IF1 KO cells. On the other hand, the selective quantification of metastatic cells showed a decrease of their number in both control and IF1 KO cell-derived xenografts upon treatment (Fig. 3C (ii)). The decreased number of metastatic cells upon treatment (Fig. 3C (i-ii)) suggested that HK affects the metastatic behavior independently of IF1.

### Honokiol treatment prevents tumor growth and colony formation in vitro

In order to analyze the mechanisms that are associated with the effects of HK on tumor masses, and thus on cells subjected to nutrient and oxygen fluctuations, colony formation in soft agar was analyzed in response to treatment.

**Fig. 4:**
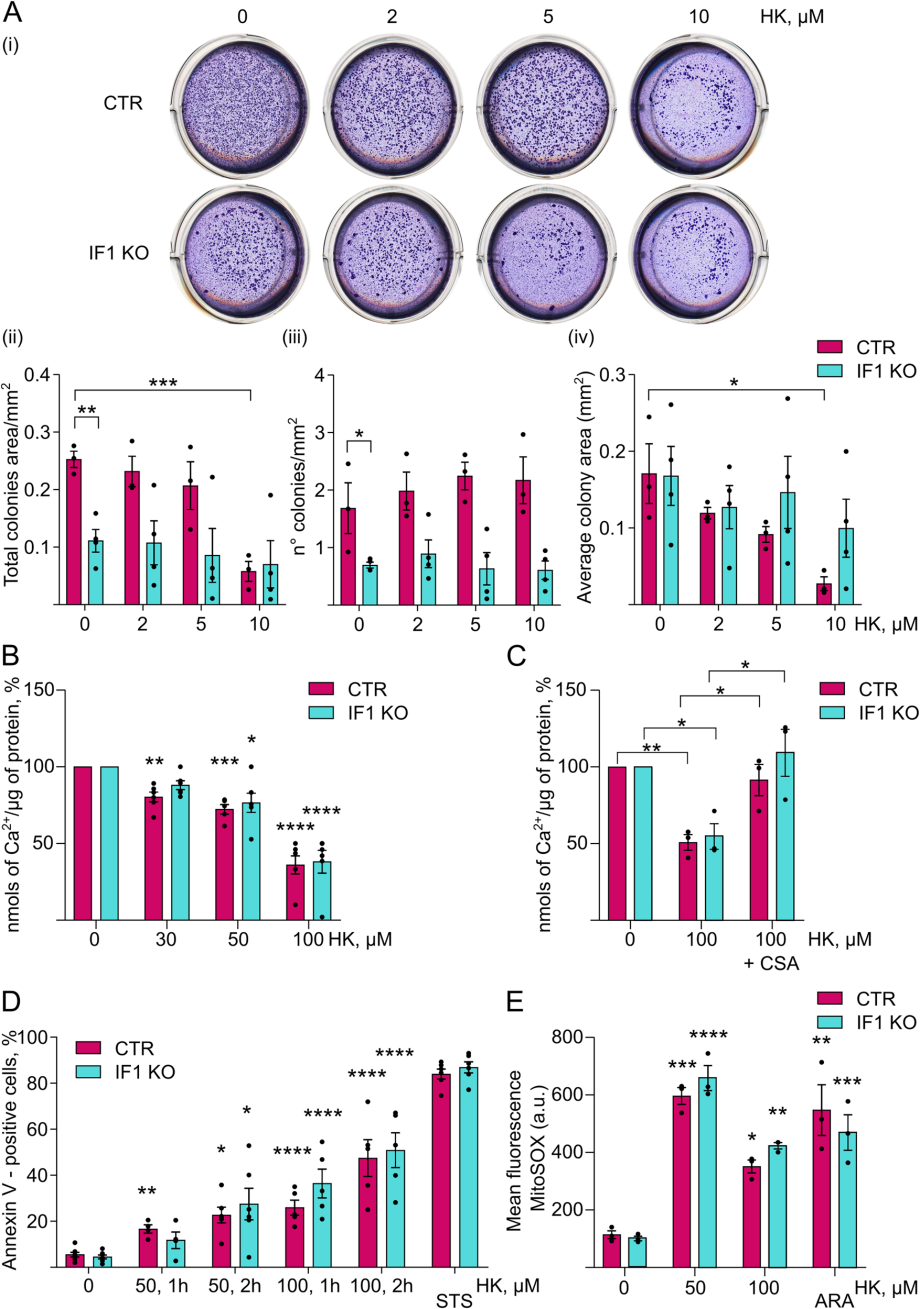
Honokiol causes a decrease of tumor growth in soft agar by promoting PTP opening and apoptosis. **A** Representative images (i) of colony formation in soft agar by CTR and IF1 KO HeLa cells treated for 15 days with 0-10 µM HK. Histograms show the mean of the total colony area/mm^2^ (ii), the number of colonies/mm^2^ (iii) and the average of colony area in mm^2^ (iv). Data are mean ± SEM of at least three independent experiments, *p ≤ 0.05, **p = 0.0029, ***p = 0.001, Student’s *t* test. **B**, **C** Calcium retention capacity is assessed in permeabilized CTR and IF1 KO HeLa cells in a buffer promoting state 3 respiration, containing the membrane-impermeable Ca^2+^ sensor, Ca^2+^ Green-5N. Histogram represents nmols of Ca^2+^ (per μg of protein) that are necessary to induce PTP opening. **B** Cells are treated with 0-100 μM HK. Data are mean (expressed as % of untreated controls) ± SEM of five independent experiments. *p = 0.0103, **p = 0.0036, ***p = 0.0002, ****p ≤ 0.0001, One-way Anova. **C** Cells are treated with/without 100 μM HK in the presence or absence of 1.6 μM CSA. Data are mean (expressed as % of untreated controls) ± SEM of three independent experiments. *p ≤ 0.05, **p = 0.0045, One-way Anova. **D** Adherent CTR and IF1 KO HeLa cells are treated with 0–100 μM HK for 1 or 2 h, or with 2 μM staurosporine for 24 h (STS), detached and incubated with the annexin V-fluorescent probe to quantify the apoptotic cells by cytofluorimetric measurements. Histogram shows the mean quantification ± SEM of apoptotic annexin V-positive cells (expressed as % of the total cell number). Data are from at least four independent experiments. *p ≤ 0.05, **p = 0.0051, ****p ≤ 0.0001, One-way Anova. **E** Adherent CTR and IF1 KO HeLa cells are treated with 0–100 μM HK for 30 min, or with 100 μM arachidonic acid for 1 h (ARA), detached for cytofluorimetric measurements. Mitochondrial superoxide anion analysis is performed by incubating cells with MitoSOX red. In the histogram, MitoSOX mean fluorescence (a.u.) is shown. Data are mean ± SEM of three independent experiments. *p = 0.0345, **p ≤ 0.01, ***p ≤ 0.001, ****p ≤ 0.0001, One-way Anova.

Control HeLa cells and their IF1 KO counterparts were studied for their colony-forming capacity in soft agar in vitro (Fig. 4A). IF1 gene ablation decreased the propensity to grow in colonies of HeLa cells, in comparison to related controls (Fig. 4A (i-ii)). Indeed, quantification of the total area occupied by tumor cell colonies, which are able to grow under stress conditions and are insensitive to contact inhibition, was significantly higher in cells with high levels of IF1, after 15 days in soft agar (Fig. 4A (ii)). The treatment with 10 µM HK for the same period of time, significantly decreased the total area and the mean area of colonies formed by control cells, but not by IF1 KO cells (Fig. 4A (i-vi)), suggesting that the compound acts on the same mechanism as IF1 in these in vitro conditions. The mechanism causing a lower colony formation in soft agar of IF1 KO and HK-treated control HeLa cells did not seem related to a block of the cell cycle (Supplementary Fig. 1A). The analysis of cell proliferation (Supplementary Fig. 1A) in the presence of increasing HK concentrations showed that HK did not significantly inhibit cell duplication in culture up to a 50 μM treatment concentration, while a 50 μM treatment concentration, prolonged for 96 h, resulted toxic on proliferation (Supplementary Fig. 1A). Moreover, cell treatment up to 10 µM HK for 72 h did not show inhibition of mitochondrial respiration in intact adherent cells (Supplementary Fig. 1B).

### Honokiol promotes permeability transition and apoptosis through a dual action on the OSCP subunit of ATP synthase

To test the hypothesis that HK might modulate the PTP in cancer cells through the OSCP subunit, we investigated its effects on Ca^2+^ retention capacity (CRC) of mitochondria derived from control and IF1 KO cells. IF1 KO HeLa mitochondria were previously shown to be more sensitive than controls to the presence of Ca^2+^ which causes PTP opening, in state 3 respiratory conditions (during oxidative phosphorylation) [55]. The difference between control and IF1 KO HeLa mitochondria was confirmed here, although the threshold of Ca^2+^ required for PTP opening are both shown as 100% in the untreated samples, for clarity (Fig. 4B, C). Acute HK treatment, at low doses (30 µM), significantly decreased the Ca^2+^ concentrations that are necessary to promote PTP opening in control mitochondria incubated in a state 3-promoting buffer (Fig. 4B). On the contrary, 30 µM HK treatment was not effective in decreasing the Ca^2+^ threshold of IF1 KO HeLa cells (Fig. 4B). Since in wild-type HeLa cells, the HK binding to the OSCP N-terminus caused the IF1 release from the OSCP subunit (Fig. 2A), it is plausible that the different change in Ca^2+^-sensitivity for PTP opening of control and IF1 KO cells, upon treatment with 30 µM HK (Fig. 4B), was due to a higher affinity of HK for the OSCP subunit N-terminal region where IF1 binds (Fig. 1D), than for the other HK binding site on the C-terminus of this subunit (Fig.1E). Increasing concentrations of HK (50 and 100 µM) were able to promote PTP opening with further decreased Ca^2+^ threshold, independently of the IF1 levels (Fig. 4B), suggesting that HK treatment above 50 µM might act on the other predicted binding site, with lower affinity (Fig.1E), on the C-terminus of the OSCP subunit. CsA, the known inhibitor of PTP opening, was able to completely inhibit the effect of HK on PTP opening, at any of the HK concentrations used up to 100 µM (Fig. 4C), suggesting the specific action of HK on the channel modulation.

The apoptotic cell death, which is activated by PTP opening and the release of mitochondrial pro-apoptotic factors, was analyzed by annexin V-staining of control and IF1 KO HeLa cells. Adherent intact cells were subjected to HK treatment for 1 or 2 h and annexin V-positive cells were counted through fluorescence detection (Fig. 4D). Treatment of cells with 50 µM HK for 1 h induced a 20% -increase of apoptosis in control cells, containing the inhibitor protein IF1, compared to untreated cells (Fig. 4D). In this condition, the IF1 KO cells did not show significant differences from the IF1 KO untreated cells (Fig. 4D). However, higher doses or longer treatments were able to promote increasing levels of apoptosis (% of annexin V-positive cells on the total number of cells) in both control and IF1 KO models, recapitulating the different affinity of HK for the two proposed binding sites on the OSCP subunit (Fig. 1D, E). Staurosporine (2 µM for 24 h of treatment) was used as a positive control of cell death in these sets of experiments (Fig. 4D). Of note, the HK concentrations that are required to activate apoptosis in intact cells were higher than the concentrations that are effective on the permeabilized cells used for the CRC analysis, suggesting that HK is permeable across the plasma membrane, but due to other possible targets, higher concentrations of the compound are needed to target mitochondria in intact cells. To assess the role of HK in promoting PTP opening through ROS generation, adherent cells were treated with 50 and 100 µM HK for 30 min and mitochondrial ROS were measured by MitoSOX fluorescent probe (Fig. 4E). Arachidonic acid treatment which is a known PTP inducer, causing mitochondrial ROS accumulation, was used as a positive control. Both 50 and 100 µM HK treatment caused a significant increase of mitochondrial ROS, although no difference was observed between control and IF1 KO cells, suggesting that the direct binding of HK to the OSCP site 1 (Fig. 1D) was more effective than ROS in causing PTP opening, thus promoting higher cell death in control than IF1 KO cells treated with 50 µM HK (Fig. 4D).

### Honokiol treatment prevents epithelial-mesenchymal transition of tumor cells in vitro

**Fig. 5:**
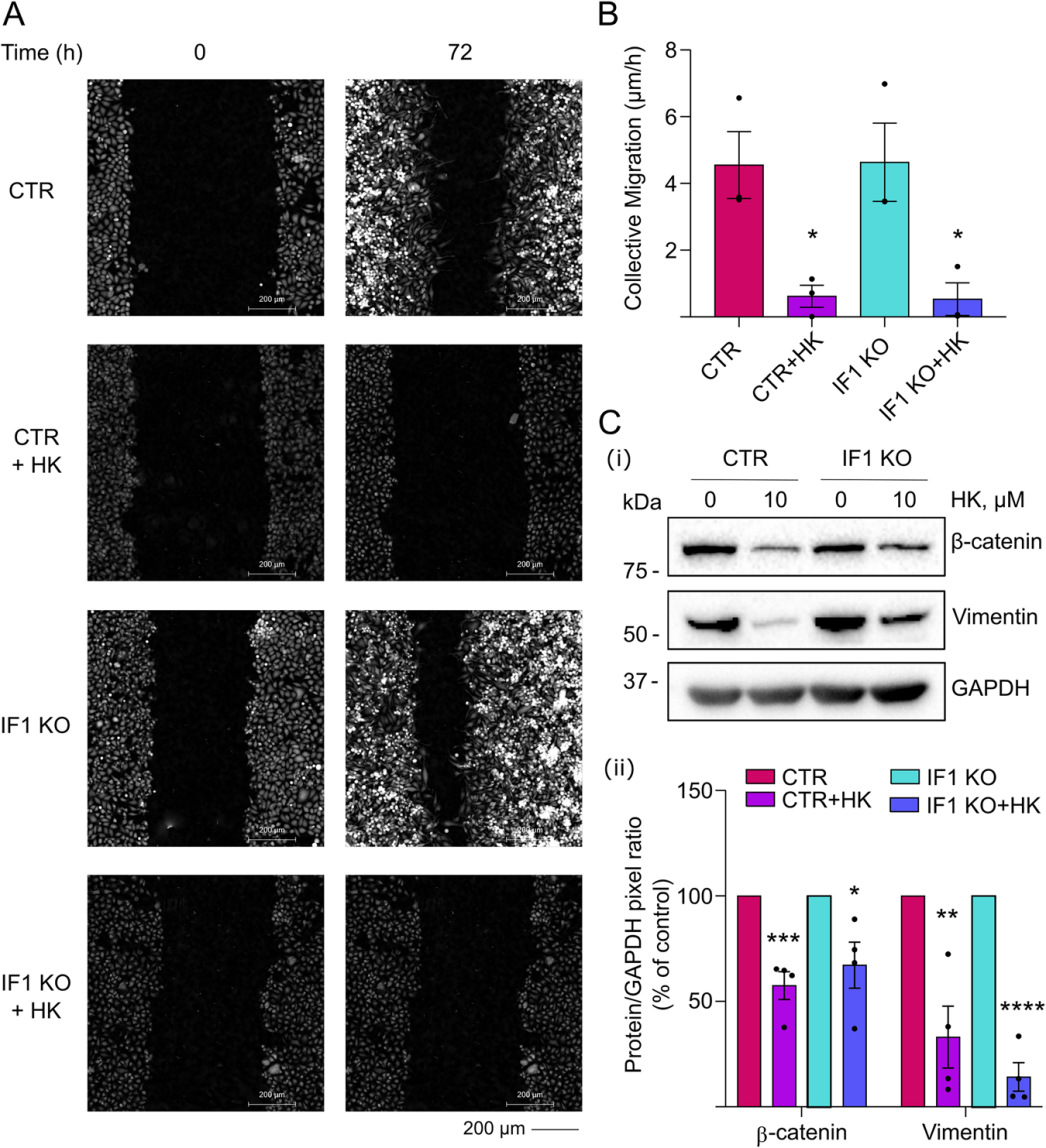
Honokiol blocks cell migration of CTR and IF1 KO HeLa models. CTR and IF1 KO HeLa cells are subjected to wound healing assay and are treated in the absence or presence of 10 µM HK. Cell migration is measured for 72 h with the Phasefocus Livecyte system. **A** Representative Quantitative Phase Images of CTR and IF1 KO HeLa cells at 0 and 72 h. Scale bar, 200 µm. **B** Histogram shows the collective (total population) migration (µm/h) of CTR and IF1 KO HeLa cells at 48 h in the absence or presence of 10 µM HK. Data are mean ± SEM of three independent experiments. *p ≤ 0.05, Student’s *t* test. **C** Western blotting shows β-catenin and vimentin proteins in lysates of CTR and IF1 KO HeLa cells, untreated or treated with 10 µM HK for 72 h in wound healing condition (i). The molecular marker is indicated on the left. Histogram (ii) shows the mean protein/GAPDH pixel ratio (expressed as % of control). Data are mean ± SEM of four independent experiments. *p = 0.024, **p = 0.004, ***p = 0.0007, ****p ≤ 0.0001, Student’s *t* test.

To test the effects of HK on cell migration, a wound healing assay was performed with HeLa cells for 72 h. Control and IF1 KO cells were not different in their wound closure rates (Fig. 5A, Supplementary Fig. 2A and Supplementary Movies 1–4), although single-cell speed of the leading cells on the front of migration appeared slightly decreased in the absence of IF1 (Supplementary Fig. 2B), as matched by the different time required for the wound-area coverage in control and IF1 KO cells (Area T1/2, Supplementary Fig. 2C). These results are in line with the observations of metastatic behavior of cells in vivo (Fig. 3B, C).

The overall collective migration, which includes cells on the front and cells that are proliferating on the back side of the monolayer area (Fig. 5B) and directionality (Supplementary Fig. 2D) did not show differences between IF1 KO and control HeLa cells. Long-time treatment (72 h) with 10 µM HK fully inhibited single-cell speed (Supplementary Fig. 2B), collective migration and caused cell dispersion, which was characterized by round directionality (Fig. 5B, Supplementary Fig. 2D). Finally, the time required for treated HeLa cells to heal the monolayer-wound was approximated to infinite in both IF1 KO and controls, due to the block of migration which was caused by HK treatment (Supplementary Fig. 2A, C). Epithelial-mesenchymal transition (EMT) markers were analyzed in lysates of cells subjected to a wound healing assay, in the absence or presence of 10 µM HK treatment (72 h) as above. HK treatment caused decreased levels of β-catenin and vimentin in both control and IF1 KO cells (Fig. 5C), as matched by the ability to migrate through a trans-well system in 72 h of untreated cells, and the decreased ability of HK-treated cells (Supplementary Fig. 2E).

### Mitochondrial adaptations under epithelial-mesenchymal transition sensitize cancer cells to honokiol treatment

**Fig. 6:**
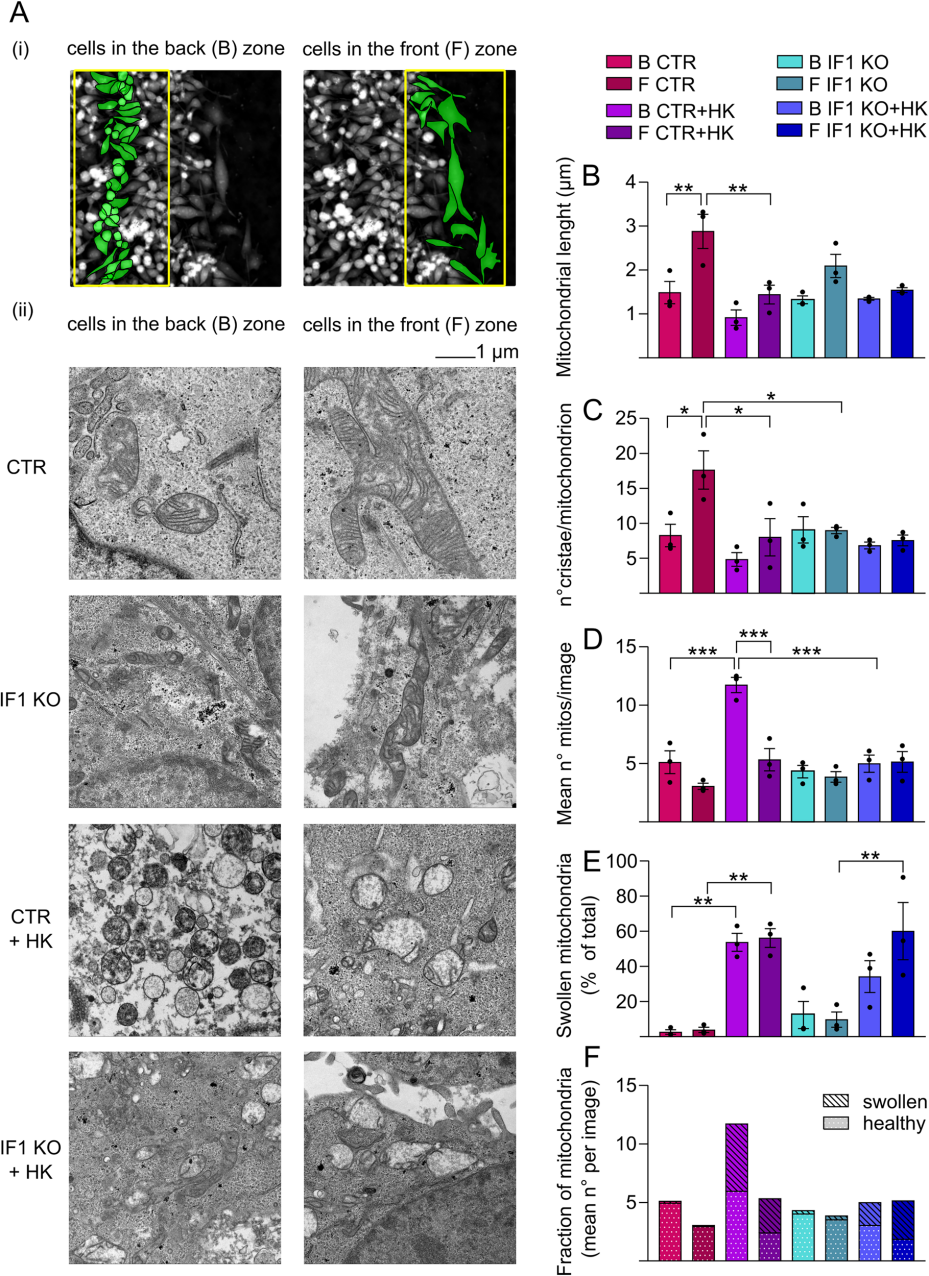
Effects of honokiol on mitochondrial morphology during cell migration. **A** Representative images of mitochondria of CTR and IF1 KO HeLa cells fixed at 72 h after wound healing assay in the absence or presence of 10 µM HK. For mitochondrial morphology analysis cells are divided in two zones (i): cells in the back (B) zone (proliferating cells) and cells in the front (F) zone (migrating cells). Representative images of transmission electron microscopy (ii) are shown of CTR and IF1 KO HeLa mitochondria in the back and front zone at 72 h. Scale bar, 1 µm. **B**–**F** Histograms show the mean mitochondrial length (µm) (**B**) the number of cristae/mitochondrion (**C**) the mean number of mitochondria per image (**D**) the number of swollen mitochondria (% of the total) (**E**) and the fractions of swollen or healthy mitochondria (mean number per image) (**F**). Data are mean ± SEM of three independent experiments. *p ≤ 0.05, **p ≤ 0.01, ***p ≤ 0.001, One-way Anova.

HeLa cell monolayers, derived from each wound healing assay (72 h) described above, were fixed and analyzed to identify the mechanisms causing a block of cell migration in the presence of HK treatment. Fixed cells were studied by transmission electron microscopy (TEM) to investigate mitochondrion and *crista* morphology. In order to discriminate between the two cell populations that (i) migrate on the front zone of the wound area, or (ii) proliferate on the back zone of the monolayers, the different regions were carefully marked in each well and recognized during the TEM analysis (Fig. 6A (i)). Cell morphology appeared different, showing elongated and larger cell structures in the front zone, or round-shaped and smaller areas in the back zone. The typical morphology, which appeared independently of the presence of IF1, is shown for controls (Fig. 6A (i)).

Mitochondria appeared larger with a longer major axis in both control and IF1 KO cells that are migrating in the front zone compared to the respective mitochondria in the back zone, although the lack of IF1 caused a less dramatic size-increase than the one observed in migrating control HeLa cells (Fig. 6A (ii), Fig. 6B). Furthermore, the number of *cristae* per mitochondrion was largely increased in migrating control HeLa cells (Fig. 6C, CTR), while their number was not changed in migrating IF1 KO cells (Fig. 6C, IF1 KO). The HK treatment for 72 h on cells subjected to wound healing assay caused unexpected effects on the rearrangements of mitochondria described. On the one hand, 10 μM HK caused an increase in the mitochondrial mass and in the number of mitochondria in control HeLa cells proliferating in the back zone, but not in IF1 KO cells (Fig. 6A (ii); Fig. 6D).

On the other hand, the HK treatment caused mitochondrial swelling, accompanied by *crista* disruption, selectively in migrating cells on the front zone, independently of the presence or absence of IF1 (Fig. 6A (ii); Fig. 6E). Overall, the mitochondrial swelling in cells treated with HK, which can be due to the sensitization to PTP opening, decreased the fraction of healthy mitochondria that remained functional during migration (front zone) in both control and IF1 KO cells, but did not affect the number of healthy mitochondria in proliferating cells in the back zone (Fig. 6F).

The protein levels from lysates derived from cells subjected to the wound healing assays were further analyzed by Western blotting (Supplementary Fig. 3). Indeed, the quantification of the oxidative phosphorylation complexes (CI, CII, CIII, CIV and CV), key mitochondrial transporters (ANT, MPC) and the biogenesis marker PGC1α, showed that a treatment with 10 μM HK for 72 h might contribute to promote biogenesis in controls, but not in IF1 KO HeLa cells (Supplementary Fig. 3A, B). This finding, although lysates derived from a mixed population of cells from both the B and the F zones, had a tendency which is in line with the increased number of mitochondria in the proliferating cells in the back zone (Fig. 6D). None difference was observed in the amount of the mitochondrial isoform of superoxide dismutase, while a mild increase of sirtuine 3 was found upon treatment of both control and IF1 KO cells (Supplementary Fig. 3B). Interestingly, in control HeLa cells the HK treatment for 72 h also caused a tendency of increased expression of proteins sensitizing mitochondria to PTP opening, such as the ATP synthase subunit c and CyPD, or a decreased expression of the inhibitor IF1, which acts on the inhibition of the mitochondrial channel (Supplementary Fig. 3C).

## Discussion

This study shows that HK interacts with the ATP synthase OSCP subunit and controls tumor growth and migration both in vivo and in vitro through PTP-dependent swelling and apoptosis.

Our in silico molecular docking simulations of HK on the OSCP protein from the recently resolved cryo-EM structure of the human ATP synthase complex [57] revealed two possible sites with high binding affinity: the first on the N-terminus, the second on the C-terminus of the OSCP subunit. The first pose falls in the same OSCP region (V60, R62, G93), which also binds IF1 in cancer cells overexpressing this protein [55, 58]. IF1 has been found to be overexpressed in many tumors. During oxidative phosphorylation, IF1 binds to the OSCP subunit of ATP synthase and promotes tumorigenesis by protecting cancer cells from PTP-dependent apoptosis [55]. In this work, the natural molecule HK was shown to disrupt the IF1-OSCP interaction through its binding to the first pose and cause a consequent sensitization to PTP opening and apoptosis of HeLa cells naturally overexpressing IF1, but not of IF1 KO counterpart. The second predicted binding site of HK on the C-terminal region of the OSCP subunit, characterized by slightly lower affinity than the first pose, falls in a region in which the OSCP subunit is in contact with the peripheral stalk of ATP synthase through the b subunit. The interaction of HK at this site, at higher concentration of compound, was found to promote PTP opening through a mechanism which is independent of IF1, but sensitizing cells to apoptosis. This HK interaction is compatible with the studies revealing the ATP synthase involvement in the PTP formation at the level of the inner mitochondrial membrane [46, 48], and with the hypothesis that the OSCP subunit might modulate the PTP through its conformational changes transmitted to the peripheral stalk and then to the membrane [29, 47]. The two poses where HK interacts are both promising to develop anti-cancer compounds due to their effect in promoting PTP opening and apoptosis. The one with higher binding affinity, at the N-terminus, is the most exposed and is available in the matrix of cancer cells that are either hydrolyzing or synthesizing ATP, as revealed by the molecular dynamic calculations of the OSCP interactions during the catalytic states 3a and 3b we have here elucidated. Of note, the residues of the OSCP subunit involved in the IF1 binding are exposed on the ATP synthase complex during catalysis, but not in state 1 or 2 when these residues are involved in interactions with other ATP synthase subunits, as previously hypothesized [58].

Through the sensitization to PTP opening and PTP-dependent apoptosis, HK is shown here to block tumor growth and metastases in a zebrafish xenograft model. It was previously shown that HK inhibits tumor growth and metastasis in U-87 MG human glioblastoma xenografts in nude mice and in immunotolerant zebrafish [3]. Moreover, HK was shown to be effective in mouse xenografts generated with a large number of other cellular models [9].

Our results, with a cervix carcinoma HeLa cell line which naturally overexpresses IF1, showed that HK inhibited tumor mass in zebrafish and colony formation in soft agar through the activation of PTP-dependent apoptosis in an IF1-dependent mechanism at the lowest treatment concentrations, suggesting its specific binding on the N-terminal site of the ATP synthase OSCP subunit. In support of the HK mechanism of action through PTP-dependent apoptosis, the HK concentrations that blocked colony formation in our study were not effective on basal mitochondrial respiration, nor on cell proliferation. Other authors found that CyPD-dependent PTP opening, but not Bcl family-activated apoptosis, is a key effect of HK in blocking breast cancer cell growth [24], in activating mTOR signaling pathway in HEK-293 cells [26], in promoting cell death in primary human acute myelogenous leukemia cells and HL60 cells in a pilot in vivo study [25].

HK in this work also blocks metastases, in a fish xenograft model, and epithelial to mesenchymal transition (EMT), in vitro. EMT is a multiple change of factors controlling cell metabolism and morphology adaptations, aimed at cancer cell transition to a more aggressive and metastatic phenotype. The complexity of cellular pathway rewiring involves a decrease of mitochondrial respiration and an increase of ROS in mitochondria. We observed that, in contrast with the current knowledge on the decrease of mitochondrial metabolism and parallel increase of glycolysis during EMT [59], in migrating HeLa cells, both with or without IF1, a huge increase in mitochondrial size, number of *cristae* and major axis length of mitochondria were observed, suggesting a high mitochondrial function requirement for cell migration, at least in this study.

HK treatment of fish caused a decrease of metastases in both xenografts injected with control and IF1 KO HeLa cells. To better understand the HK mechanism of action, the same cell lines used for the in vivo studies were analyzed through TEM microscopy at the end of a wound healing assay. Mitochondria of HK-treated migrating cells appeared mostly swollen, possibly due to PTP opening, independently of the content of IF1. It is plausible that the increase of mitochondrial ROS, which is a key process during the EMT, but is also a positive modulator of PT [60], resulted in lethality in combination with the HK treatment, which further sensitizes PTP opening. On the other hand, the HK treatment did not cause swelling, but biogenesis, in the cells that are proliferating in culture in the back zone, and do not migrate, although the lack of the inhibitor IF1 limited biogenesis. Western blotting analysis of HeLa lysates derived from wound healing assay, and corresponding to the cells that are both in the front and the back zones in TEM images, showed an increase of respiratory complexes, pyruvate and adenine nucleotide carriers, and ATP synthase subunits, paralleled by an increase in PGC1α. These changes were found in control HeLa cells, matching the higher increase of mitochondrial size or number, in the front or back zone, respectively, compared to IF1 KO HeLa cells. Our results, showing a tendency of increase in SIRT3 and PGC1α, are in line with the previous finding that HK treatment activates SIRT3 and PGC1α in hypertrophic failing hearts, although initially characterized by a low level of PGC1α [27].

The role of HK in inhibiting mitochondrial respiration has been proposed in an initiation model of lung squamous cell carcinoma in vivo and in cell lines [23] and in orthotopic lung tumor xenografts developing brain metastases in vivo [61]. Both studies suggested that HK triggers the generation of ROS through the inhibition of mitochondrial function. Although our colony formation analysis and wound healing assay were conducted at low doses of HK treatment that did not interfere with mitochondrial respiration in basal conditions, we cannot exclude that the effects in promoting PTP-dependent swelling and apoptosis, blocking colony formation and migration, might be further exacerbated by ROS of mitochondrial origin.

In conclusion, we have shown that honokiol is a promising anti-cancer molecule by limiting tumor growth in tumors that upregulate the mitochondrial IF1 inhibitor protein, but it also achieves anti-metastatic effects independently of IF1. As shown by the two HK binding sites, the ATP synthase OSCP subunit is a novel, reliable target in mitochondria to develop new anti-cancer strategies acting on PTP-dependent apoptosis.

## Materials and methods

### In silico molecular docking

The three-dimensional (3D) structure of human OSCP from the recently resolved cryo-EM structure of the human ATP synthase complex (PDB ID: 8H9V, chain O, residues 23–213) was used for molecular docking calculations [57]. Before docking, the OSCP structure was prepared in Chimera [62] by removing other chains from the ATP synthase complex, non-standard amino acids, and water molecules. The residues were protonated by adding missing hydrogen atoms at pH 7.4.

The 3D conformer of HK was downloaded from PubChem (PubChem CID 72303; https://pubchem.ncbi.nlm.nih.gov/compound/Honokiol). The ligand was prepared by adding hydrogen atoms at pH 7,4 and optimizing the geometry using Avogadro software (http://avogadro.cc). All relevant dihedral angles were set to be flexible during docking calculations.

Molecular docking calculations were carried out using AutoDock Vina 1.1.2 [56, 63]. The OSCP and ligand files were converted to pdbqt format using OpenBabel and served as input for AutoDock Vina. To comprehensively sample potential binding sites of HK on the OSCP structure, “blind” docking calculations were performed. AutoDock Tool was used to set a grid centered on the protein’s center of mass, with a deliberately large search volume. AutoDock Vina generated nine molecular poses, sorted by binding affinity, with clusters located in different spatial regions.

The interactions between HK and OSCP at the two docking sites were analyzed using RING v4.0 (https://ring.biocomputingup.it/ [64]). OSCP complexes with the two HK poses were used as input to build residue interaction networks (RINs) to detect non-covalent inter-chain interactions. “Closest nodes” and “strict” and “relaxed” thresholds were used as parameters for constructing the RINs. RING v4.0 was also used to identify inter-chain interactions between OSCP and other subunits in the ATP synthase complex, using the four rotational states of ATP synthase structure - states 1, 2, 3a, 3b (PDB ID: 8H9S, 8H9T, 8H9U, 8H9V) - as input. Residues involved in interactions in the ATP synthase complex and with the experimental IF1 binding sites from [55] were mapped onto the OSCP structure and compared with residues forming the potential HK binding sites (RING-Pymol plugin [65]).

### Stable knocking out of IF1 protein

Stable IF1 knocking out (IF1 KO) was obtained by HeLa transfection with two different guides (G1 and G2) cloned into the lentiCRISPRv2 plasmid and targeting the human IF1 gene. Sequences for the guides (G1: 5′- CGGACGTGGCTTGGCGTGTG -3′ and G2: 5′-CAGTCCGAGAATGTCGACCG -3′) were found by using the CRISPR design tool and were generated following the manufacturer’s procedures. Briefly, oligonucleotide pairs were annealed and cloned into lentiCRISPRv2 plasmid (Addgene, Watertown, MA, USA #52961) and co-transfected into HEK-293T cells, with the three packaging plasmids pMDLg/pRRE, pRSV-Rev and pMD2.G, for viral production. HeLa cells were infected by standard methods, with both the recombinant virus containing G1 and the recombinant virus containing G2. Scrambled lentiCRISPRv2GFP (Addgene #51760) was used as a control (CTR). Transfected HeLa cells were selected with 0.8 μg/ml puromycin and analyzed through Western blotting.

### Experimental models

HeLa cells were obtained from the American Tissue Culture Collection (ATCC). Wild-type, CTR and IF1 KO HeLa cells were cultured in Dulbecco’s modified Eagle’s medium (DMEM, Thermo Fisher Scientific, Waltham, MA, USA), supplemented with fetal bovine serum (FBS, 10% v/v, Thermo Fisher Scientific), glutamine (4 mM, Thermo Fisher Scientific), penicillin and streptomycin (1% v/v, Thermo Fisher Scientific) at 37 °C with 5% CO_2_. Cells were free of mycoplasma contamination, as routinely tested.

### In vivo and in vitro treatment

HK treatments were defined upon dose titration at the time of treatments that were needed by different experimental assays. Acute treatments (1–2 h) of adherent cells with 50 and 100 μM HK were required to cause PTP-dependent cell death without cell toxicity. Adherent cells were incubated with these HK concentrations for 30 min for PLA and ROS analyses. Acute treatment (minutes) of mitochondria or permeabilized cells showed that lower HK concentrations (30 μM) were effective in causing PTP opening, possibly due to other unspecific targets that in intact cells sequestrate HK molecules during diffusion to mitochondria. Long-term HK treatments were defined upon titration of HK concentrations and selecting conditions that did not affect cell viability and mitochondrial respiration for 72 h. In vivo treatments were carried out up to 1 μM HK, doses that did not cause embryo toxicity and preserved fish viability.

### Zebrafish xenografts

Zebrafish experiments were performed at the Zebrafish Facility of the University of Padova (Italy). Zebrafish embryos were obtained by natural mating of adult fish (Tuebingen line). Embryos and adults were raised and maintained according to standard (ZFIN) protocols. For the injection, CTR and IF1 KO HeLa cells were detached with trypsin, resuspended in serum-containing cell culture medium, washed with PBS, and stained with the fluorescent dye Vybrant DiI (1 mg/mL, catalog number V22885, Thermo Fisher Scientific). The stained cells were injected into zebrafish embryos at 2 days post-fertilization, previously anesthetized with a solution of 160 mg/L tricaine. The cells were injected into the yolk as a single droplet (200 μm diameter, about 100 cells per embryo) using a World Precision Instrument microinjector. One day after injection, the fish were assessed for successful yolk injection and 0.1 or 1 μM HK and 1‰ DMSO (for control) were added once in fish water (total volume of 20 ml). Fish were monitored until 4 days post injection. The cell-derived fluorescent tumor mass was imaged at 6 dpf under fluorescence microscopy (M165-FC microscope with DFC7000T digital camera, Leica Microsystems, Milan, Italy) and quantified with the Measurements option of the Volocity 6.0 software (Perkin Elmer, Milan, Italy).

### Immunoprecipitation of OSCP subunit and ATP synthase

Freshly prepared mitochondria from wild-type HeLa cells were obtained after cell homogenization with a glass-Teflon potter as previously described [66] then the immunoprecipitation of OSCP subunit or ATP synthase was performed. Freshly prepared mitochondria (500 mg) were resuspended in 500 μl of a buffer promoting ATP synthesis (0.1 M glucose, 80 mM KCl, 10 mM Mops-Tris, 5 mM succinate-Tris, 4 mM MgCl_2_, 1 mM Pi-Tris, 0.5 mM NADP^+^, 0.4 mM ADP, 50 μM Pi, P5-di (adenosine-5′) pentaphosphate, 10 μM EGTA, 4 U/ml glucose-6-phosphate dehydrogenase, and 3 U/ml hexokinase, pH 7.4) and incubated with or without 30 and 50 µM of HK (Sigma Aldrich, St. Louis, MO, USA) for 10 min at room temperature. Mitochondria were then centrifuged at 8 000 × g for 10 min at 4 °C.

To perform the immunoprecipitation of the OSCP subunit, mitochondria were resuspended in 400 μl of the IP buffer (50 mM NaCl, 30 mM Tris-HCl, 2 mM EGTA-Tris, 5 mM aminocaproic acid, 0.005% (w/v) BSA, 0.5% (v/v) Triton X-100, 0.25% (w/v) SDS, pH 7.4), supplemented with 2 μg of anti-OSCP antibody (Abcam, Cambridge, UK) and incubated for 2 h at 4 °C under wheel rotation. Finally, 30 μL dynabeads protein G (Thermo Fisher Scientific) were added, and samples were incubated overnight at 4 °C under wheel rotation. The day after, the supernatant was collected (named Extracts in Fig. 2 and Original blots), beads were washed three times with IP buffer (15 mM Tris-HCl, 2 mM EDTA-Tris, 1.8 mM EGTA-Tris, 0.005% (w/v) BSA and 0.5% (w/v) Triton X-100, pH 7.4) and proteins were eluted with sample buffer (NuPAGE™ LDS Sample buffer, Thermo Fisher Scientific, supplemented with 12.5% v/v β-mercaptoethanol), indicated as Eluates in Fig. 2 and Original blots. The collected fractions were subjected to SDS-PAGE followed by Western blotting to detect OSCP subunit and IF1. For the immunoprecipitation of ATP synthase, mitochondria were resuspended in 50 μL of 0.75 M aminocaproic acid, 50 mM Bis-Tris, pH 7.0, and solubilized with digitonin (1% w/v, Sigma Aldrich). After centrifugation at 100 000 × g for 25 min at 4 °C, the supernatant was collected. Extracted proteins were supplemented with 10 μl of anti-complex V monoclonal antibody covalently linked to protein G-sepharose beads (ATP synthase immunocapture kit, Abcam), and incubated overnight at 4 °C under wheel rotation. Beads were centrifuged at 500 × g for 5 min at 4 °C, and washed twice for 5 min in a solution of 0.05% (w/v) n-dodecyl b-D-maltoside (Sigma Aldrich) in PBS. For the elution, beads were incubated three times with sample buffer for 15 min, and the collected fractions (eluates) were subjected to SDS-PAGE, together with their corresponding digitonin extracts, followed by Western blotting to detect β subunit and IF1.

### Proximity ligation assay

Proximity ligation assay (PLA) was carried out using the manufacturer’s instructions provided for Duolink In Situ Red Starter kit (DUO92101 Sigma), modified as follows. Sterile coverslips were placed in a 24-well plate. CTR HeLa cells were seeded at 50×10^3^ cells/well on coverslips. The day after seeding, cells were incubated with 50 μM HK for 30 min and fixed with 2% (w/v) paraformaldehyde, permeabilized with 0,15% (v/v) Triton X-100 for 5 min at room temperature and washed with PBS. Cells were incubated with Duolink Blocking Solution for 30 min at room temperature under gentle shaking. According to the manufacturer’s instructions, two primary antibodies were used in combination in each coverslip, in order to detect possible interactions occurring between two proteins in close proximity. The primary antibodies applied in couple derived from different species (i.e. mouse (M) or rabbit (R)) and were: anti-ATPB ab14730; anti-ATPase IF1 ab223779; anti-ATPase IF1 ab110277, from Abcam; anti-OSCP SC74786, from Santa Cruz Biotechnology, Inc. (Dallas, TX, USA), diluted 1:30 in Duolink Antibody Diluent for incubation overnight, at 4 °C into a humidity chamber. The day after, secondary antibodies conjugated with oligonucleotides (PLA probe anti-mouse MINUS and PLA probe anti-rabbit PLUS) were incubated with samples for 1,5 h at 37 °C. Ligation and amplification were performed according to the manufacturer’s instructions. The amplification reaction mix was incubated for 2 h at 37 °C in the humidity chamber. After washing, coverslips were mounted with Duolink In Situ Mounting Medium containing DAPI fluorescent probe for nuclei detection. Red dots detecting protein interactions were analyzed through a Leica Stellaris 8—DMi8 CS equipped confocal microscope with a HC PL APO CS2 63x/1.40 OIL objective.

### Calcium retention capacity

For the CRC assay, external mitochondrial Ca^2+^ was measured by Ca^2+^ Green-5N fluorescence using a Tecan Infinite® 200 PRO (Tecan Trading AG, Männedorf, Switzerland) plate reader in permeabilized cells. Cells were detached with trypsin, centrifuged at 1000 × *g* for 5 min, and washed twice with PBS. The pellet was resuspended in KCl-based medium (130 mM KCl, 10 mM Mops-Tris, pH 7.4) supplemented with 1 mM EGTA-Tris and 180 μg/ml digitonin, and incubated for 20 min on ice. Cells were then diluted 1:10 in KCl medium containing 10 μM EGTA-Tris, and centrifuged at 1000 × *g* in a refrigerated centrifuge (4 °C) for 5 min. The final pellet containing permeabilized cells, was then resuspended, at the concentration of 10^6^ × ml^−1^, in a buffer promoting ATP synthesis (s3), supplemented with 5 mM succinate-Tris, 1 mM Pi-Tris, 0.5 μM Ca^2+^ Green-5N and treated with or without 30, 50, and 100 µM HK to a final volume of 0.2 ml. In the case of CsA treatment, permeabilized cells were incubated 5 min on ice with 1.6 µM CsA in the aforementioned buffer, containing 5 mM Pi-Tris, before the addition of HK. For CRC measurements, sequential 5 μM CaCl_2_ pulses were added to cells and Ca^2+^ Green-5N fluorescence was measured. Calcium (nmols) taken up by mitochondria were normalized for the µg of protein in the sample.

### Apoptosis

CTR and IF1 KO HeLa cells were seeded at 200,000 cells/well in a 12-well tissue culture plate. The day after seeding, cells were incubated with 50 and 100 μM HK in bicarbonate- and phenol red-free Hanks’ balanced salt solution (HBSS) containing 10 mM HEPES, pH 7.4 at 37 °C. After 1 and 2 h of treatment, cells were harvested with trypsin and counted. Cells incubated only with HBSS, containing 10 mM HEPES pH 7.4, at 37 °C for 2 h were harvested with trypsin and counted, for control. Staurosporine (2 μM) treatment was used as a positive control for cell death, and was incubated in the same conditions above for 24 h. Cell death was assessed by the Muse cell analyzer (Millipore, Burlington, MA, USA) using Muse Annexin V and Dead Cell Kit (Luminex Flow Cytometry & Imaging, Austin, TX, USA), following the manufacturer’s instructions.

### Mitochondrial reactive oxygen species (ROS)

The mitochondrial superoxide anions were analyzed by loading HeLa cells with MitoSOX^TM^ Red dye (Thermo Fisher Scientific) as previously reported [67]. CTR and IF1 KO HeLa cells were seeded at 200,000 cells/well in a 12-well tissue culture plate. The day after seeding, cells were incubated with 50 or 100 μM HK for 30 min or with 100 μM arachidonic acid for 1 h (ARA, Sigma Aldrich) in bicarbonate- and phenol red-free Hanks’ balanced salt solution (HBSS) containing 10 mM HEPES, pH 7.4 at 37 °C. Then the cells were washed with PBS, harvested with trypsin and the cell fluorescence intensity was assessed by the Muse cell analyzer (Millipore).

### Oxygen consumption rate

CTR and IF1 KO HeLa cells were seeded at 2500 cells/well in a 96-well tissue culture plate in the cultured medium supplemented with 0, 2, 5, 10 µM HK. The OCR of adherent cells was monitored for 72 h using the Resipher System (Lucid Scientific, Atlanta, GA, USA). The system’s patented dynamic optical oxygen sensors provide the highest sensitivity without disturbing cells. During the experiment, cells were kept at 37 °C in a 5% CO_2_ humidified incubator.

### Colony formation in soft agar

For the soft agar assay, a phenol red-free DMEM medium, supplemented with 4mM glutamine, 1mM pyruvate, penicillin, and streptomycin, was used. CTR and IF1 KO HeLa cells were seeded at 25,000 cells/well in a 12 well tissue culture plate in a 0.6% (w/v) agar matrix, between a bottom layer composed of 1% (w/v) agar matrix and a top layer of complete DMEM supplemented with 1% (v/v) FBS. The day after seeding, cells were treated with 0, 2, 5, and 10 µM HK and grown for 15 days at 37 °C in a 5% CO_2_ humidified incubator, changing the treatment medium every 2 days. After 15 days, colonies were washed in PBS, stained with a 0.005% (w/v) Crystal Violet solution (Sigma Aldrich) and analyzed with ImageJ software.

### Cell migration by wound healing assay

CTR and IF1 KO HeLa cells were seeded at 120,000 cells/well in a 24-well plate in order to reach 90% of confluence. After 24 h a scratch area was created using a pipette tip, followed by rinsing with PBS. Cells where then monitored under treatment with or without 10 µM HK in the cell culture medium. Cell migration was followed for 72 h with the Livecyte system (Phase Focus Ltd, Sheffield, UK), acquiring images every hour. Effects on wound closure were examined using Quantitative Phase Imaging (QPI). The high contrast images and the Cell Analysis Toolbox® (CAT) software generates measurements of the time taken for the area of the scratch to halve (area T1/2), mean rate of the closure of the scratch (collective migration), cell speed and cell direction of the leading-edge cells. Analysis was carried out using the Phase Focus CAT® software.

### Cell migration by trans-well assay

A 6-well plate containing 8-mm pore size ThinCert® cell culture inserts (Greiner Bio-One, Kremsmünster, Austria) was used to evaluate the migration of tumor cells.

CTR and IF1 KO HeLa cells were seeded at 1.2 × 10^6^ in the upper chamber in 1 ml of serum-free medium. In the lower chamber, 3 ml of medium supplemented with 10% FBS in the presence or absence of 10 μM HK were added. After 72 h, cells that had migrated through the pores were imaged and counted by the EVOS M5000 Imaging System inverted microscope (Thermo Fisher Scientific).

### Transmission Electron Microscopy

CTR and IF1 KO HeLa cells treated or untreated with 10 µM HK after wound healing assay (72 h) were fixed for TEM analysis. Samples were post-fixed with 1% (v/v) osmium tetroxide in 0.1 M sodium cacodylate buffer for 1 h. After three water washes, samples were dehydrated in a graded ethanol series and embedded in an epoxy resin (Sigma Aldrich). Ultrathin sections (60–70 nm) were obtained with an Ultrotome V (LKB) ultramicrotome, counterstained with uranyl acetate and lead citrate, and viewed with a Tecnai G2 (FEI, Hillsboro, OR, USA) transmission electron microscope operating at 100 kV. Sections were cut in parallel to cell monolayer, in order to allow recognition by TEM observations of empty areas where cells are migrating, and confluent areas where cells are proliferating. Images were captured with a Veleta (Olympus Soft Imaging System, Tokyo, Japan) digital camera. Images were analyzed by counting the total number of mitochondria, *cristae* per mitochondrion, mitochondrial length, healthy and swollen mitochondria.

### Growth curve analysis

CTR and IF1 KO HeLa cells were seeded at 15 000 cells/well in a 24-well tissue culture plate in the cultured medium supplemented with 0, 2, 5, 10, 30, 50 µM HK. The treatment was changed every second day. Cells were grown for 96 h, at 37 °C in a 5% CO_2_ humidified incubator. After 24, 48, 72, and 96 h, cells were harvested with trypsin and counted.

### Western blotting analysis

CTR an IF1 KO HeLa cells were seeded in a 60 mm dish in order to reach 90% of confluence. After 24 h, a scratch area was created using a scraper, followed by rinsing with PBS. Cells were then treated with or without 10µM HK in the cell culture medium and allowed to grow for 72h. At 72 h cell lysates were performed as it follows, cells (10 × 10^6^) were harvested with trypsin, kept on ice for 20 min in 0.15 ml of a buffer containing 150 mM NaCl, 20 mM Tris, 5 mM EDTA-Tris, pH 7.4 with the addition of 1% (v/v) Triton X-100, 10% (v/v) glycerol and protease inhibitor cocktail (Merck, Darmstadt, Germany). Cell extracts were cleared by centrifugation at 18 000 × g for 20 min, 4 °C. Sample buffer was added to supernatants, and samples were separated by polyacrylamide gel (NuPAGE™, 12% Bis-Tris, Thermo Fisher Scientific) electrophoresis and transferred to nitrocellulose membranes. Blocking was performed with a PBS solution containing 5% (w/v) non-fat dry milk (AppliChem, Darmstadt, Germany). Antibodies for β-catenin and vimentin were from Selleck Chemicals LLC (Houston, TX, USA), OXPHOS (OXPHOS Human WB Antibody Cocktail), CyPD, IF1, ANT3, SOD2 and for β, c and OSCP subunits were from Abcam, antibodies for PGC1α, SIRT3, MPC1 and GAPDH were from Cell Signaling (Danvers, MA, USA). Band pixels of each replicate are normalized on band pixels of their proper loading control (β or OSCP subunit, GAPDH).

### Quantification and statistical analysis

Unless otherwise stated in the figure legends, each experiment was repeated at least three times. Data are presented as mean ± SEM. *P* values indicated in the figures are calculated with GraphPad, Student’s *t* test or one-way ANOVA are applied (*represents *p ≤ 0.05, **p ≤ 0.01, ***p ≤ 0.001, ****p ≤ 0.001). The variance between the groups that are compared is similar. Western blotting band intensities were analyzed using ChemiDoc MP system equipped with the ImageLab software (Bio-Rad, Hercules, CA, USA) or ImageJ software, TEM images were analyzed with the ImageJ software, while cell apoptosis measurements were analyzed using the Muse cell analyzer (Millipore) and MuseSoft Analysis and Flowing software. GraphPad and Inkscape software were used to create the figures.

## Supplementary information

Supplementary Table 1

Supplementary Table 2

Supplementary Figure 1

Supplementary Figure 2

Supplementary Figure 3

Supplementary Movie 1

Supplementary Movie 2

Supplementary Movie 3

Supplementary Movie 4

Original blots

## Data Availability

All constructs and information will be made available to the scientific community upon request.
