# Supplementary material for: Honokiol blocks tumor development and metastasis through mitochondrion-targeted effects
Source: Cell Death Dis. 2026 Jan 30;17(1):186. doi: 10.1038/s41419-026-08441-6 (PMC12877151; doi:10.1038/s41419-026-08441-6)
Supplement: Supplementary file 1 — Supplementary Table 1 [file 41419_2026_8441_MOESM1_ESM.docx]

**Supplementary Table 1. Interactions of OSCP with HK in the two poses with higher affinity.** OSCP complexes with the two HK poses with higher binding affinity were used as input to build residue interaction networks (OSCP-mod1 and OSCP-mod2) to detect non-covalent inter-chain interactions. Less and more permissive thresholds, indicated as “strict” or “relaxed”, are used as parameters for constructing the residue interaction networks (RING v4.0, <https://ring.biocomputingup.it/>). VDW is van der Waals interaction, MC and SC indicate main or side chain from which aminoacidic atoms belong, respectively. Bond distance is in Å. Atom 1 and 2 refers to Nodeld 1 and 2, respectively.

| OSCP-mod1, relaxed thresholds |  |  |  |  |  |
| --- | --- | --- | --- | --- | --- |
| NodeId 1 (HK) | Interaction | NodeId 2 (OSCP) | Distance (Å) | Atom 1 | Atom 2 |
| A:1:_:LIG | VDW:LIG_MC | O:58:_:PRO | 3.013 | **O2** | **O** |
| A:1:_:LIG | VDW:LIG_MC | O:59:_:TYR | 3.158 | **O2** | C |
| A:1:_:LIG | VDW:LIG_MC | O:60:_:VAL | 3.290 | C8 | **O** |
| A:1:_:LIG | VDW:LIG_SC | O:62:_:ARG | 2.831 | C12 | 2HH2 |
| A:1:_:LIG | VDW:LIG_SC | O:62:_:ARG | 2.853 | C17 | HD2 |
| A:1:_:LIG | VDW:LIG_SC | O:62:_:ARG | 3.410 | C7 | CB |
| A:1:_:LIG | VDW:LIG_SC | O:65:_:LYS | 2.943 | C14 | HB3 |
| A:1:_:LIG | VDW:LIG_SC | O:65:_:LYS | 3.495 | C15 | CB |
| A:1:_:LIG | VDW:LIG_SC | O:90:_:ALA | 2.940 | C15 | HB3 |
| A:1:_:LIG | VDW:LIG_SC | O:93:_:GLY | 2.339 | **H1** | HA2 |
| OSCP-mod1,  strict thresholds |  |  |  |  |  |
| NodeId 1 (HK) | Interaction | NodeId 2 (OSCP) | Distance (Å) | Atom 1 | Atom 2 |
| A:1:_:LIG | VDW:LIG_MC | O:58:_:PRO | 3.013 | **O2** | O |
| A:1:_:LIG | VDW:LIG_SC | O:62:_:ARG | 3.410 | C7 | CB |
| A:1:_:LIG | VDW:LIG_SC | O:65:_:LYS | 3.400 | C14 | CD |
| A:1:_:LIG | VDW:LIG_MC | O:93:_:GLY | 2.871 | **H1** | CA |
| OSCP-mod2, relaxed thresholds |  |  |  |  |  |
| NodeId 1 (OSCP) | Interaction | NodeId 2 (HK) | Distance (Å) | Atom 1 | Atom 2 |
| O:141:_:PHE | VDW:SC_LIG | A:1:_:LIG | 2.893 | HE2 | C18 |
| O:141:_:PHE | VDW:SC_LIG | A:1:_:LIG | 2.914 | HB3 | C12 |
| O:141:_:PHE | VDW:SC_LIG | A:1:_:LIG | 3.488 | CB | C4 |
| O:169:_:LYS | VDW:SC_LIG | A:1:_:LIG | 2.857 | HE2 | C14 |
| O:169:_:LYS | VDW:SC_LIG | A:1:_:LIG | 2.981 | HG3 | C8 |
| O:169:_:LYS | VDW:SC_LIG | A:1:_:LIG | 2.986 | H2 | C15 |
| O:169:_:LYS | VDW:SC_LIG | A:1:_:LIG | 3.434 | CB | C17 |
| O:171:_:VAL | VDW:SC_LIG | A:1:_:LIG | 2.872 | HG13 | C18 |
| OSCP-mod2,  strict thresholds |  |  |  |  |  |
| NodeId 1 (OSCP) | Interaction | NodeId 2 (HK) | Distance (Å) | Atom 1 | Atom 2 |
| O:141:_:PHE | VDW:SC_LIG | A:1:_:LIG | 2.893 | HE2 | C18 |
| O:141:_:PHE | VDW:SC_LIG | A:1:_:LIG | 2.914 | HB3 | C12 |
| O:169:_:LYS | VDW:SC_LIG | A:1:_:LIG | 3.434 | CB | C17 |
| O:171:_:VAL | VDW:SC_LIG | A:1:_:LIG | 2.872 | HG13 | C18 |
