# Supplementary material for: Honokiol blocks tumor development and metastasis through mitochondrion-targeted effects
Source: Cell Death Dis. 2026 Jan 30;17(1):186. doi: 10.1038/s41419-026-08441-6 (PMC12877151; doi:10.1038/s41419-026-08441-6)
Supplement: Supplementary file 2 — Supplementary Table 2 [file 41419_2026_8441_MOESM2_ESM.docx]

**Supplementary Table 2. Interactions of OSCP with ATP synthase subunits in different catalytic states** (**1, 2, 3a, 3b** according to Lai et al., 2023). OSCP (chain O) complexes with ATP synthase subunits (chains A, B, C for α subunit; E, F for β; K for b; L for F6) are shown (RING v4.0, <https://ring.biocomputingup.it/>). VDW is van der Waals interaction, HBOND is hydrogen bond; PICATION is π-cation bond, PIPISTACK is π-π stacking, IONIC is halogen bond. In bold type, the interactions with high energy. MC and SC indicate main or side chain from which aminoacidic atoms belong, respectively. In yellow, the OSCP residues binding to IF1 are shown. In green, the OSCP residues predicted to bind HK in the first pose (OSCP-mod1). In orange, the OSCP residues predicted to bind HK in the second pose (OSCP-mod2).

| State 1 |  |  |
| --- | --- | --- |
| NodeId 1 (ATP synthase subunit) | Interaction | NodeId 2 (OSCP) |
| A:10:_:SER | VDW:SC_SC | O:4:_:LEU |
| A:12:_:LEU | VDW:SC_SC | O:18:_:TYR |
| A:13:_:GLU | VDW:MC_SC | O:21:_:ALA |
| A:13:_:GLU | VDW:SC_SC | O:25:_:ALA |
| A:15:_:ARG | VDW:SC_SC | O:88:_:LEU |
| A:15:_:ARG | **HBOND:SC_SC** | O:91:_:GLU |
| A:15:_:ARG | VDW:SC_SC | O:91:_:GLU |
| A:15:_:ARG | VDW:SC_SC | O:91:_:GLU |
| A:16:_:ILE | VDW:SC_SC | O:22:_:LEU |
| A:16:_:ILE | **HBOND:MC_SC** | O:84:_:ASN |
| A:16:_:ILE | VDW:MC_SC | O:84:_:ASN |
| A:17:_:LEU | VDW:SC_SC | O:29:_:GLN |
| A:18:_:GLY | VDW:SC_SC | O:84:_:ASN |
| A:3:_:THR | VDW:MC_SC | O:14:_:ILE |
| A:7:_:GLU | **HBOND:SC_SC** | O:18:_:TYR |
| A:7:_:GLU | VDW:SC_SC | O:18:_:TYR |
| A:7:_:GLU | VDW:SC_SC | O:92:_:ASN |
| B:30:_:ARG | **PICATION:SC_SC** | O:59:_:TYR |
| B:30:_:ARG | VDW:SC_SC | O:59:_:TYR |
| B:32:_:LEU | VDW:SC_SC | O:58:_:PRO |
| B:42:_:HIS | **PIPISTACK:SC_SC** | O:59:_:TYR |
| C:11:_:ILE | VDW:SC_SC | O:186:_:MET |
| C:16:_:ILE | VDW:SC_SC | O:186:_:MET |
| C:23:_:VAL | VDW:SC_SC | O:173:_:MET |
| C:23:_:VAL | VDW:SC_SC | O:173:_:MET |
| C:25:_:LEU | VDW:SC_SC | O:169:_:LYS |
| C:26:_:GLU | VDW:MC_MC | O:169:_:LYS |
| C:26:_:GLU | HBOND:MC_MC | O:170:_:TYR |
| C:26:_:GLU | VDW:SC_MC | O:170:_:TYR |
| C:28:_:THR | **HBOND:SC_SC** | O:165:_:ARG |
| C:28:_:THR | VDW:SC_MC | O:168:_:GLU |
| C:45:_:ARG | **HBOND:MC_SC** | O:168:_:GLU |
| K:174:_:ILE | VDW:SC_SC | O:187:_:ARG |
| K:178:_:GLU | VDW:SC_MC | O:183:_:GLY |
| K:200:_:ASP | VDW:MC_SC | O:173:_:MET |
| K:201:_:LEU | VDW:SC_SC | O:162:_:MET |
| K:205:_:ALA | VDW:MC_SC | O:137:_:VAL |
|  |  |  |

| State 2 |  |  |
| --- | --- | --- |
| NodeId 1 (ATP synthase subunit) | Interaction | NodeId 2 (OSCP) |
| A:30:_:ARG | VDW:SC_SC | O:59:_:TYR |
| A:30:_:ARG | VDW:SC_SC | O:59:_:TYR |
| A:42:_:HIS | **PIPISTACK:SC_SC** | O:59:_:TYR |
| B:15:_:ARG | **HBOND:SC_MC** | O:185:_:ALA |
| B:15:_:ARG | VDW:SC_SC | O:186:_:MET |
| B:15:_:ARG | **HBOND:SC_MC** | O:188:_:GLU |
| B:15:_:ARG | **HBOND:SC_MC** | O:188:_:GLU |
| B:15:_:ARG | VDW:SC_MC | O:188:_:GLU |
| B:20:_:ASP | **HBOND:MC_SC** | O:181:_:LYS |
| B:20:_:ASP | VDW:MC_SC | O:181:_:LYS |
| B:21:_:THR | **HBOND:MC_SC** | O:178:_:LYS |
| B:22:_:SER | VDW:MC_SC | O:181:_:LYS |
| B:26:_:GLU | VDW:MC_MC | O:169:_:LYS |
| B:26:_:GLU | **HBOND:MC_MC** | O:170:_:TYR |
| B:68:_:PRO | VDW:SC_SC | O:12:_:TYR |
| B:68:_:PRO | VDW:MC_SC | O:12:_:TYR |
| C:12:_:LEU | VDW:SC_SC | O:18:_:TYR |
| C:12:_:LEU | VDW:SC_SC | O:88:_:LEU |
| C:13:_:GLU | VDW:MC_SC | O:21:_:ALA |
| C:15:_:ARG | VDW:SC_SC | O:88:_:LEU |
| C:16:_:ILE | VDW:MC_SC | O:84:_:ASN |
| C:16:_:ILE | VDW:MC_SC | O:84:_:ASN |
| C:16:_:ILE | VDW:SC_SC | O:84:_:ASN |
| C:16:_:ILE | VDW:SC_SC | O:85:_:LEU |
| C:7:_:GLU | VDW:SC_SC | O:14:_:ILE |
| C:7:_:GLU | VDW:MC_SC | O:17:_:ARG |
| F:15:_:ARG | VDW:SC_SC | O:6:_:ARG |
| F:29:_:ASP | VDW:MC_SC | O:3:_:LYS |
| F:29:_:ASP | **IONIC:SC_SC** | O:6:_:ARG |
| F:29:_:ASP | VDW:SC_SC | O:6:_:ARG |
| K:174:_:ILE | VDW:SC_MC | O:186:_:MET |
| K:174:_:ILE | VDW:SC_SC | O:187:_:ARG |
| K:174:_:ILE | VDW:SC_SC | O:187:_:ARG |
| K:181:_:VAL | VDW:SC_SC | O:179:_:ILE |
| K:197:_:CYS | VDW:MC_SC | O:162:_:MET |
| K:197:_:CYS | VDW:MC_SC | O:162:_:MET |
| K:204:_:LEU | VDW:MC_SC | O:141:_:PHE |
| L:42:_:LEU | VDW:SC_SC | O:178:_:LYS |

| State 3a |  |  |
| --- | --- | --- |
| NodeId 1 (ATP synthase subunit) | Interaction | NodeId 2 (OSCP) |
| A:25:_:LEU | VDW:SC_SC | O:169:_:LYS |
| A:25:_:LEU | VDW:SC_SC | O:169:_:LYS |
| A:26:_:GLU | HBOND:MC_MC | O:170:_:TYR |
| A:27:_:GLU | VDW:SC_MC | O:168:_:GLU |
| A:27:_:GLU | **IONIC:SC_SC** | O:169:_:LYS |
| A:69:_:ASP | VDW:SC_SC | O:12:_:TYR |
| C:30:_:ARG | VDW:SC_SC | O:59:_:TYR |
| E:15:_:ARG | VDW:SC_SC | O:10:_:GLN |
| E:29:_:ASP | VDW:MC_SC | O:3:_:LYS |
| E:58:_:GLU | VDW:SC_SC | O:17:_:ARG |
| E:59:_:SER | VDW:SC_SC | O:6:_:ARG |

| state 3b |  |  |
| --- | --- | --- |
| NodeId 1 (ATP synthase subunit) | Interaction | NodeId 2 (OSCP) |
| A:25:_:LEU | VDW:SC_SC | O:169:_:LYS |
| A:25:_:LEU | VDW:SC_SC | O:169:_:LYS |
| A:25:_:LEU | VDW:SC_SC | O:171:_:VAL |
| A:26:_:GLU | **HBOND:MC_MC** | O:170:_:TYR |
| A:43:_:GLY | VDW:MC_SC | O:168:_:GLU |
| A:45:_:ARG | VDW:SC_SC | O:12:_:TYR |
| A:45:_:ARG | **HBOND:MC_SC** | O:168:_:GLU |
| A:45:_:ARG | VDW:MC_SC | O:168:_:GLU |
| A:87:_:ILE | VDW:SC_SC | O:170:_:TYR |
| C:30:_:ARG | VDW:SC_SC | O:58:_:PRO |
| C:42:_:HIS | VDW:SC_SC | O:58:_:PRO |
| F:30:_:GLU | **HBOND:SC_SC** | O:61:_:LYS |
| F:30:_:GLU | **HBOND:SC_SC** | O:61:_:LYS |
| F:30:_:GLU | **IONIC:SC_SC** | O:61:_:LYS |
| F:30:_:GLU | VDW:SC_SC | O:61:_:LYS |
| K:174:_:ILE | VDW:SC_SC | O:186:_:MET |
| K:200:_:ASP | VDW:SC_SC | O:162:_:MET |
| K:207:_:LYS | VDW:SC_SC | O:141:_:PHE |
|  |  |  |
