# Supplementary figures and images for: Honokiol blocks tumor development and metastasis through mitochondrion-targeted effects
Source: Cell Death Dis. 2026 Jan 30;17(1):186. doi: 10.1038/s41419-026-08441-6 (PMC12877151; doi:10.1038/s41419-026-08441-6)

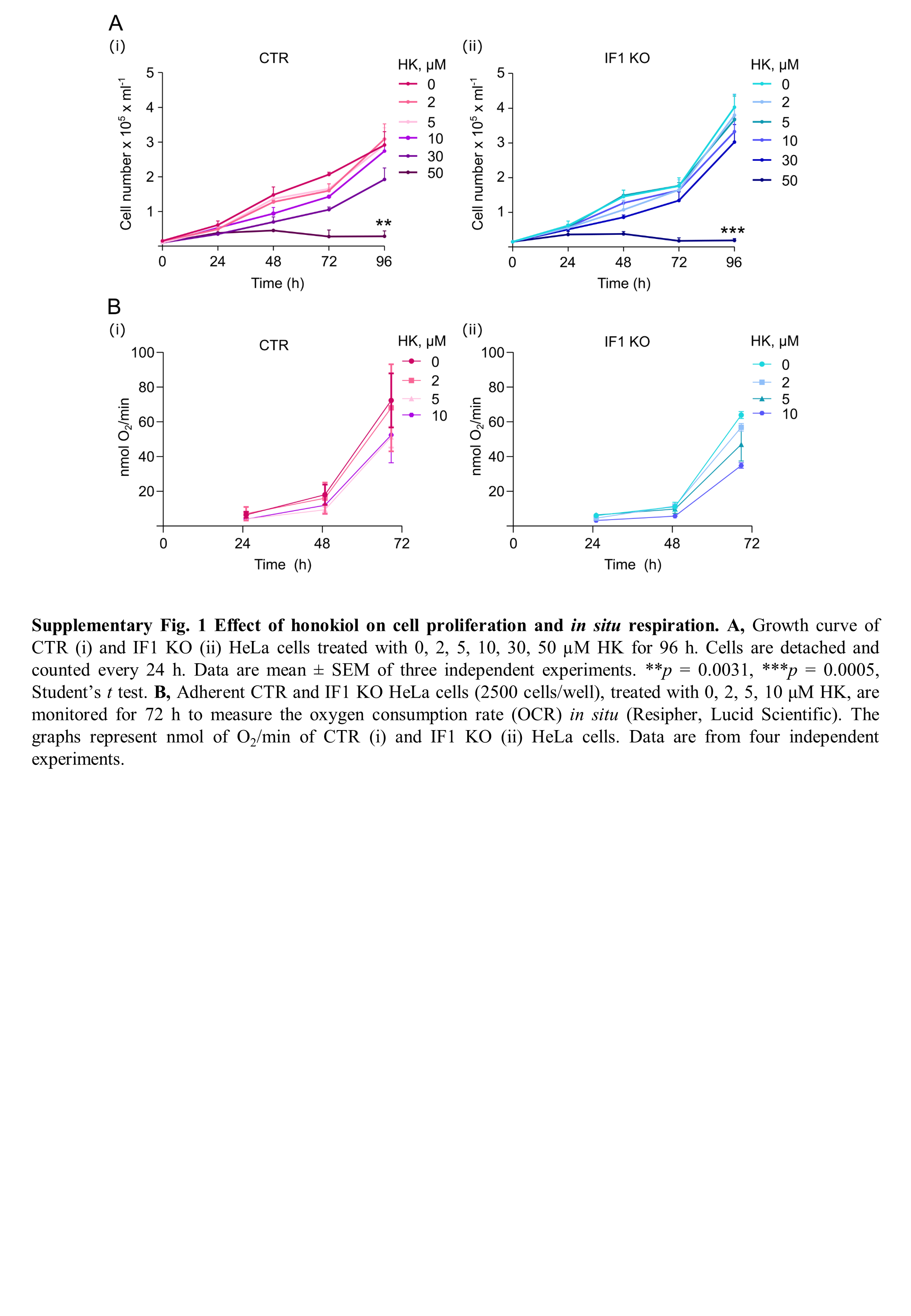

Supplement: Supplementary file 3 — Supplementary Figure 1 [file 41419_2026_8441_MOESM3_ESM.tif]

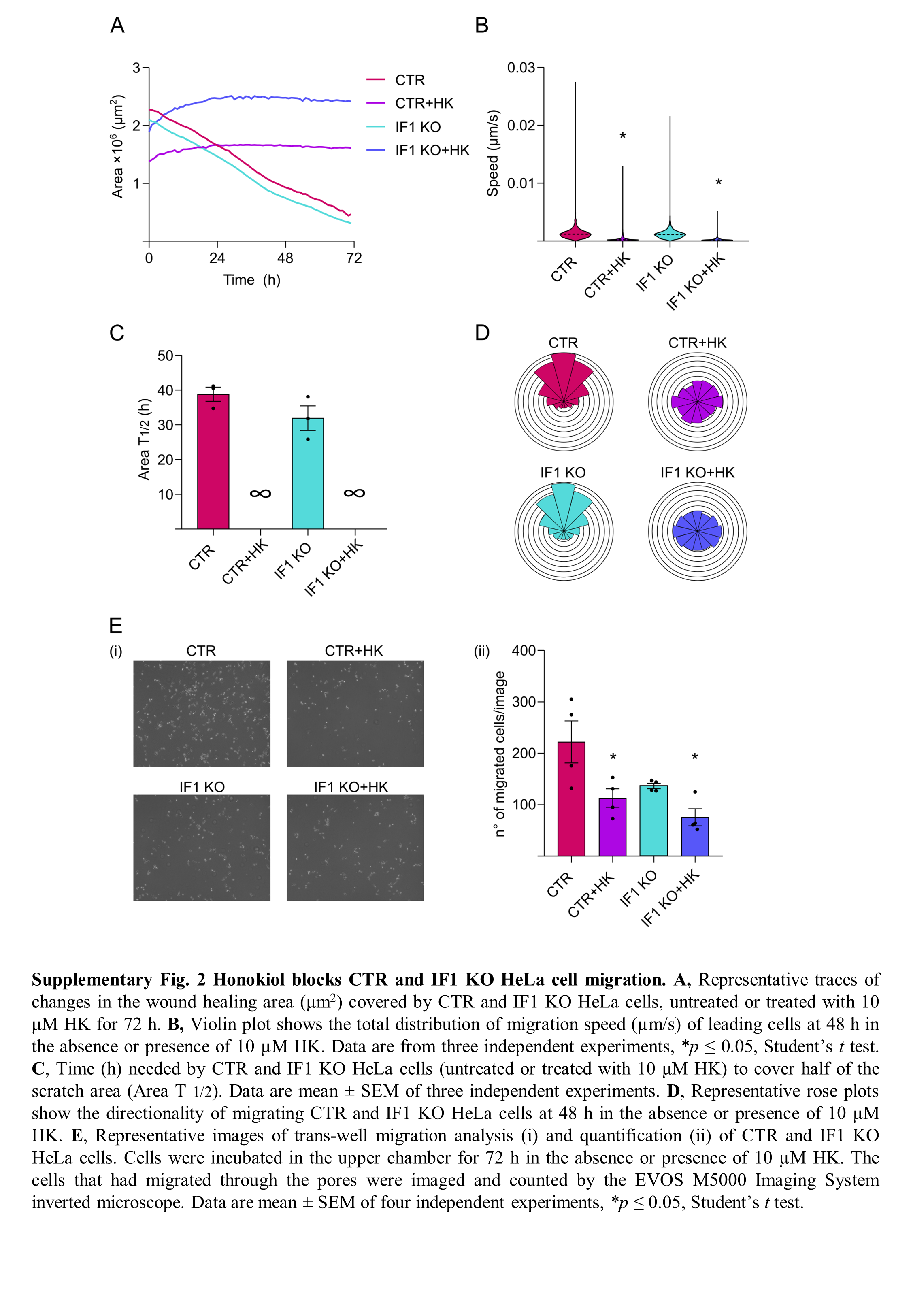

Supplement: Supplementary file 4 — Supplementary Figure 2 [file 41419_2026_8441_MOESM4_ESM.tif]

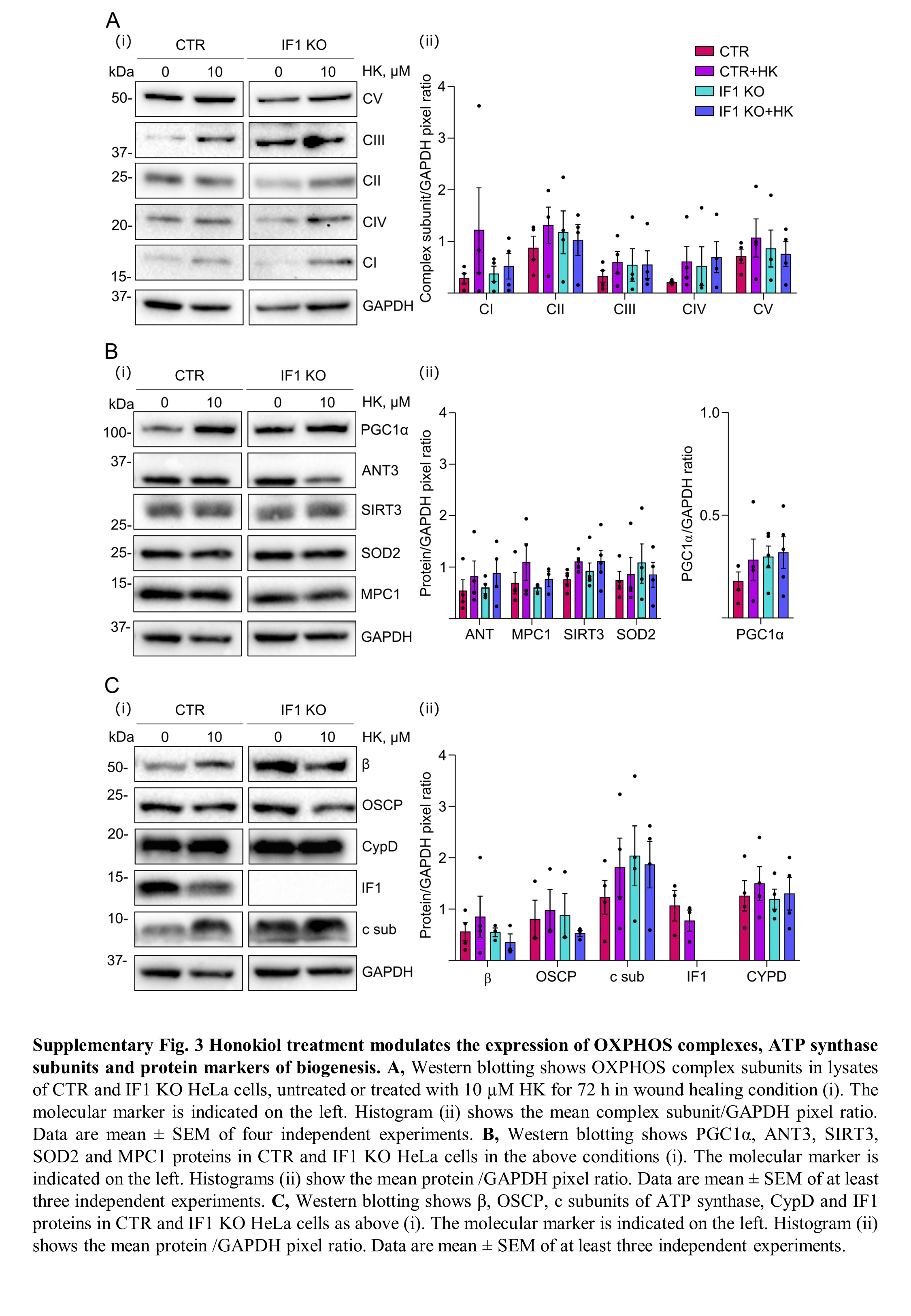

Supplement: Supplementary file 5 — Supplementary Figure 3 [file 41419_2026_8441_MOESM5_ESM.tif]
