# Supplementary material for: Honokiol blocks tumor development and metastasis through mitochondrion-targeted effects
Source: Cell Death Dis. 2026 Jan 30;17(1):186. doi: 10.1038/s41419-026-08441-6 (PMC12877151; doi:10.1038/s41419-026-08441-6)

FIGURE 1A

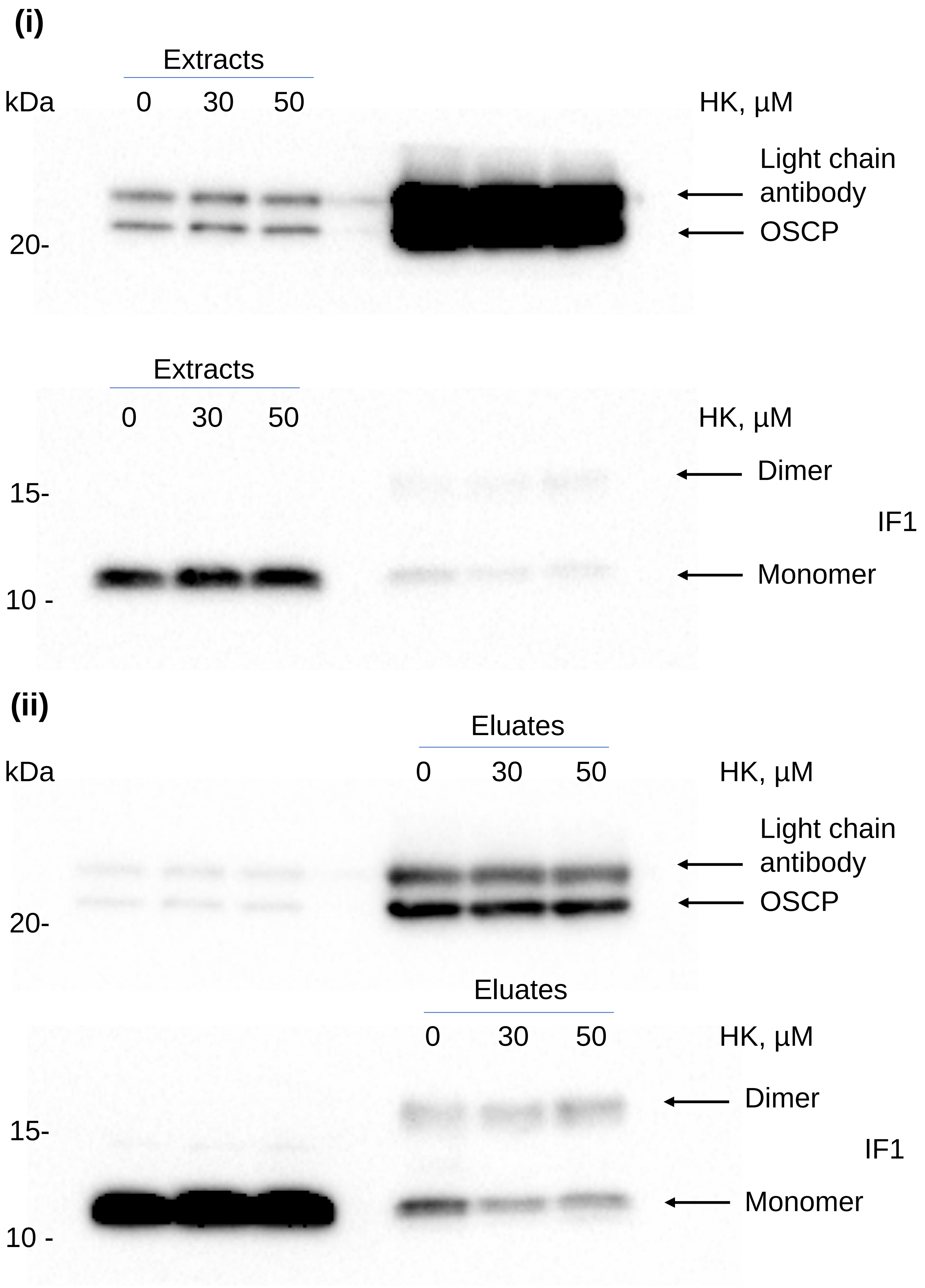
(i)
Extracts
kDa
0
30
50
HK, µM
Light chain antibody
OSCP
20-
Extracts
0
30
50
HK, µM
Dimer
15-
IF1
Monomer
10 -
(ii)
Eluates
kDa
0
30
50
HK, µM
Light chain antibody
OSCP
20-
Eluates
0
30
50
HK, µM
Dimer
15-
IF1
Monomer
10 -

FIGURE 1B

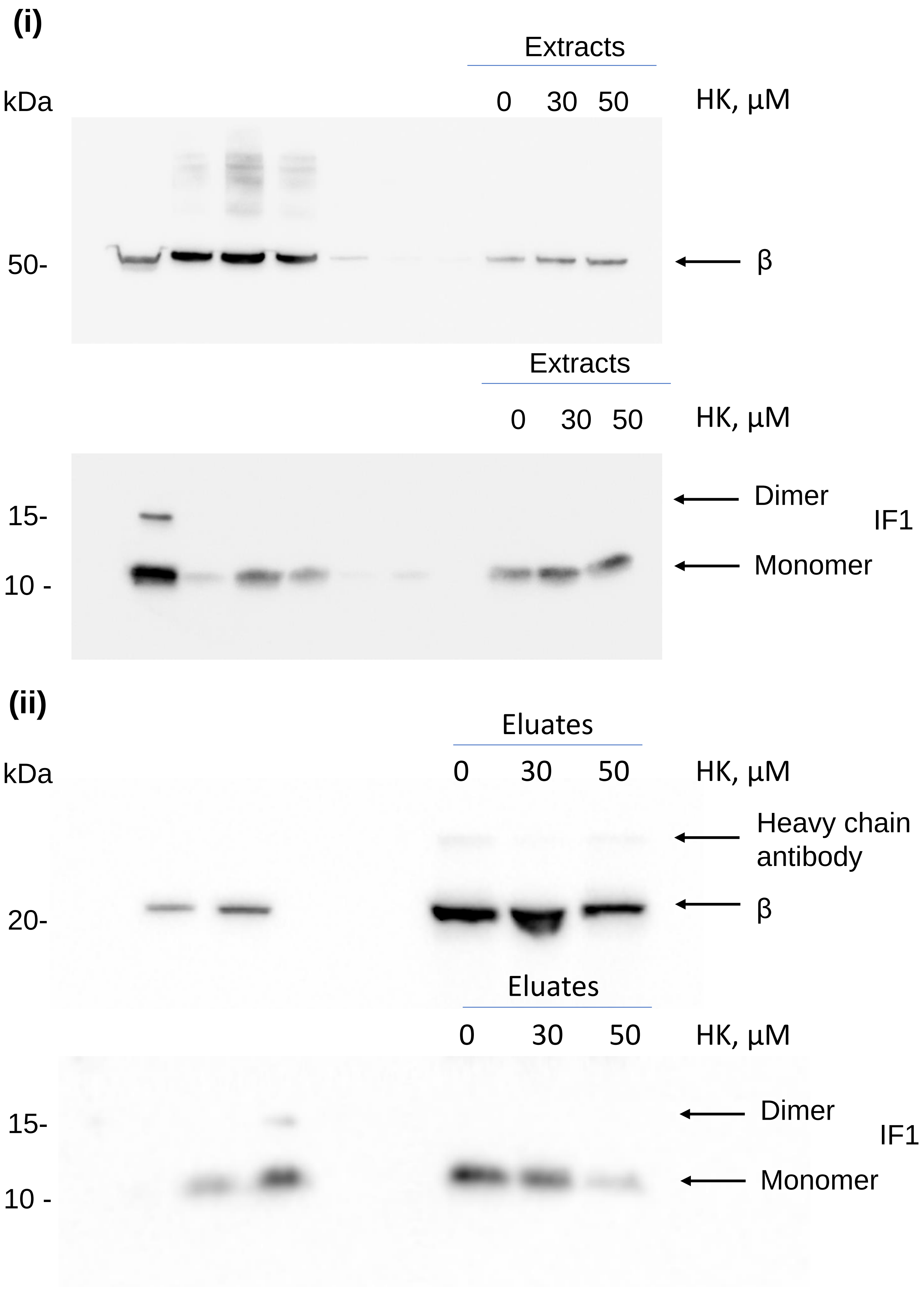
(i)
Extracts
kDa
0 30 50
HK, µM
50-
β
Extracts
0 30 50
HK, µM
15-
10 -
Dimer
Monomer
IF1
(ii)
Eluates
kDa
0 30 50
HK, µM
Heavy chain antibody
20-
β
Eluates
0 30 50
HK, µM
15-
10 -
Dimer
Monomer
IF1

FIGURE 5C

(i)

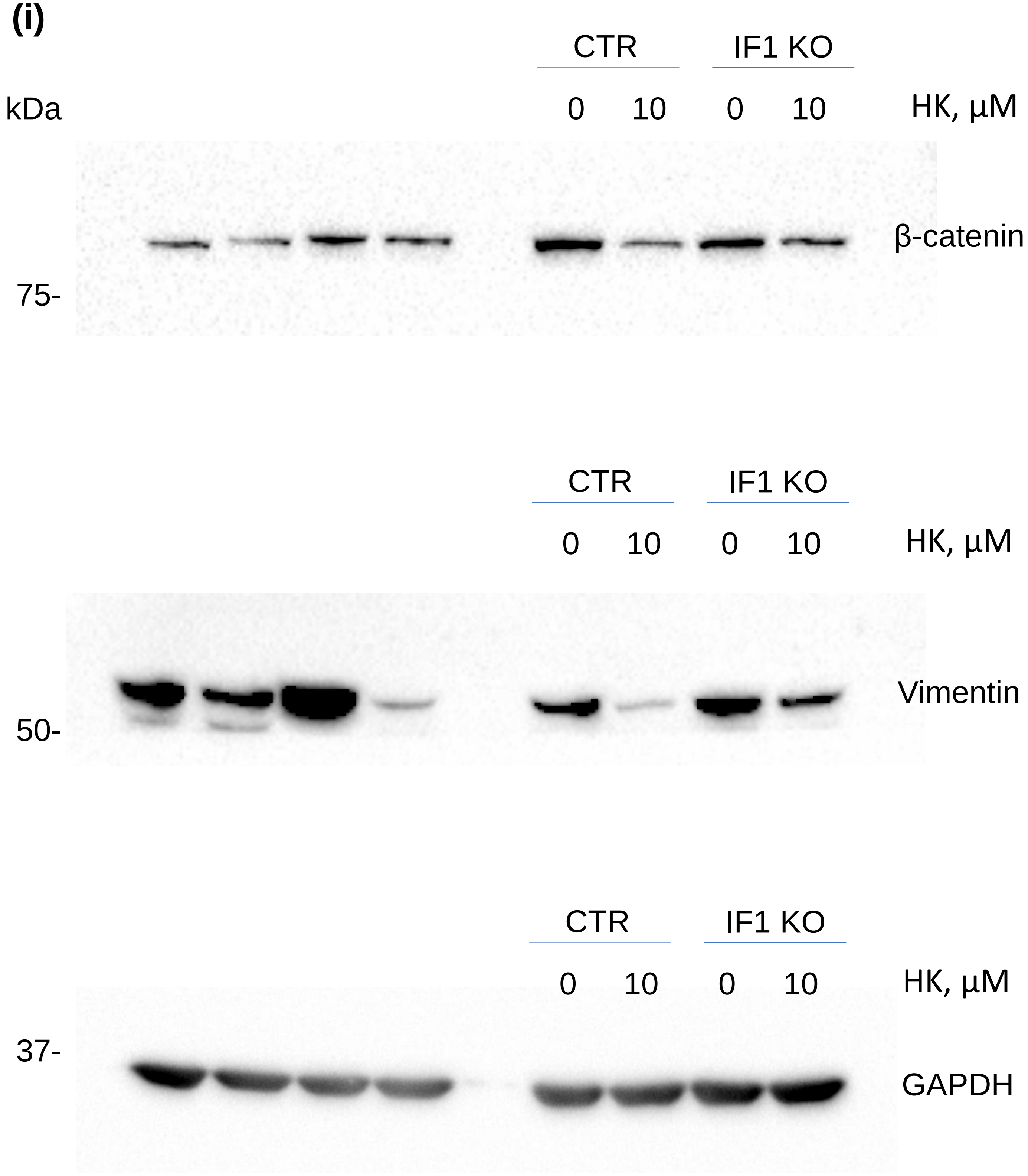
CTR
IF1 KO
kDa
0
10
0
10
HK, μM
β-catenin
75-
CTR
IF1 KO
0
10
0
10
HK, μM
Vimentin
50-
CTR
IF1 KO
0
10
0
10
HK, μM
37-
GAPDH

SUPPLEMENTARY FIG. 3A

(i)

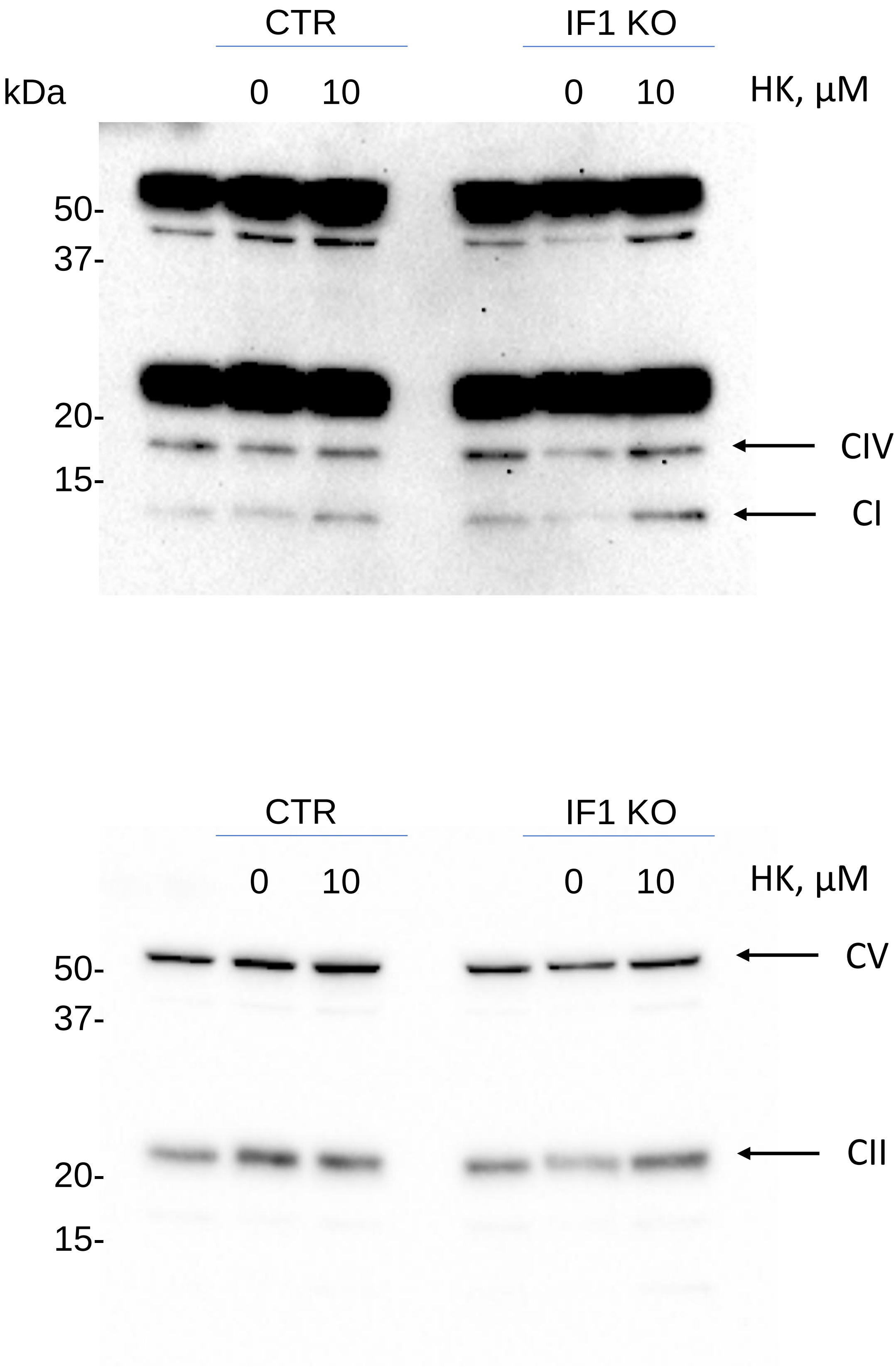

SUPPLEMENTARY FIG. 3A

(i)

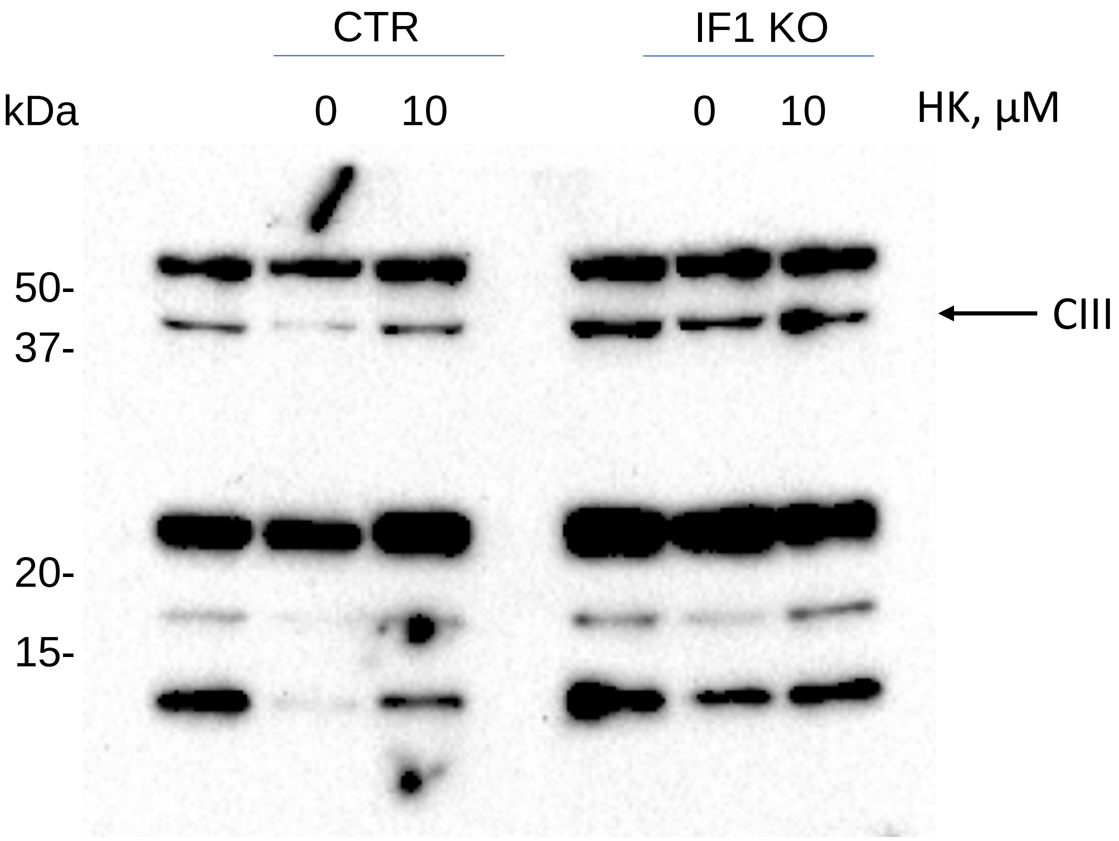

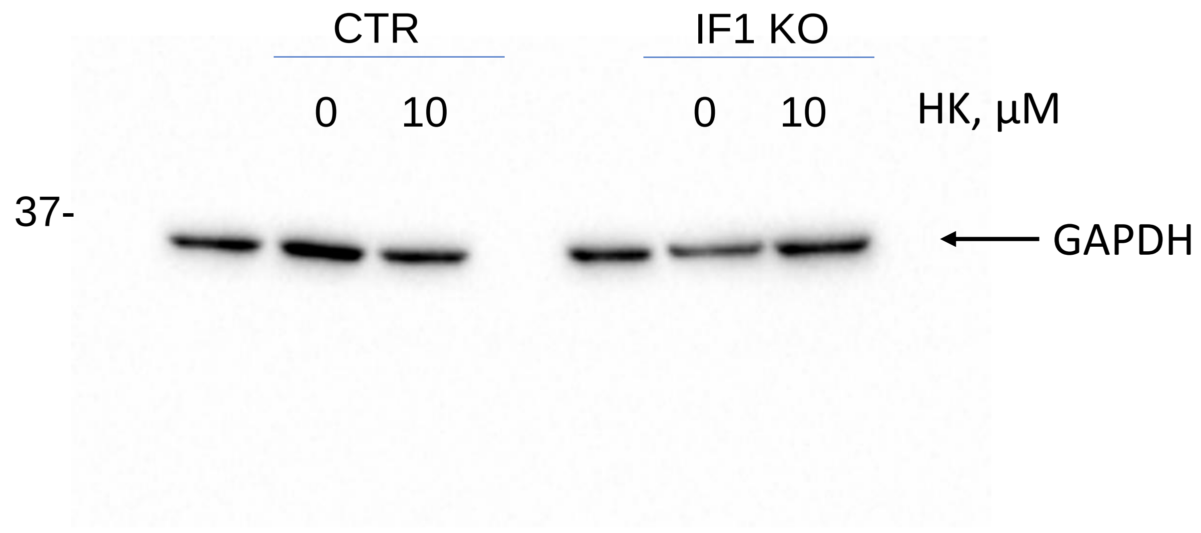

SUPPLEMENTARY FIG. 3B

(i)

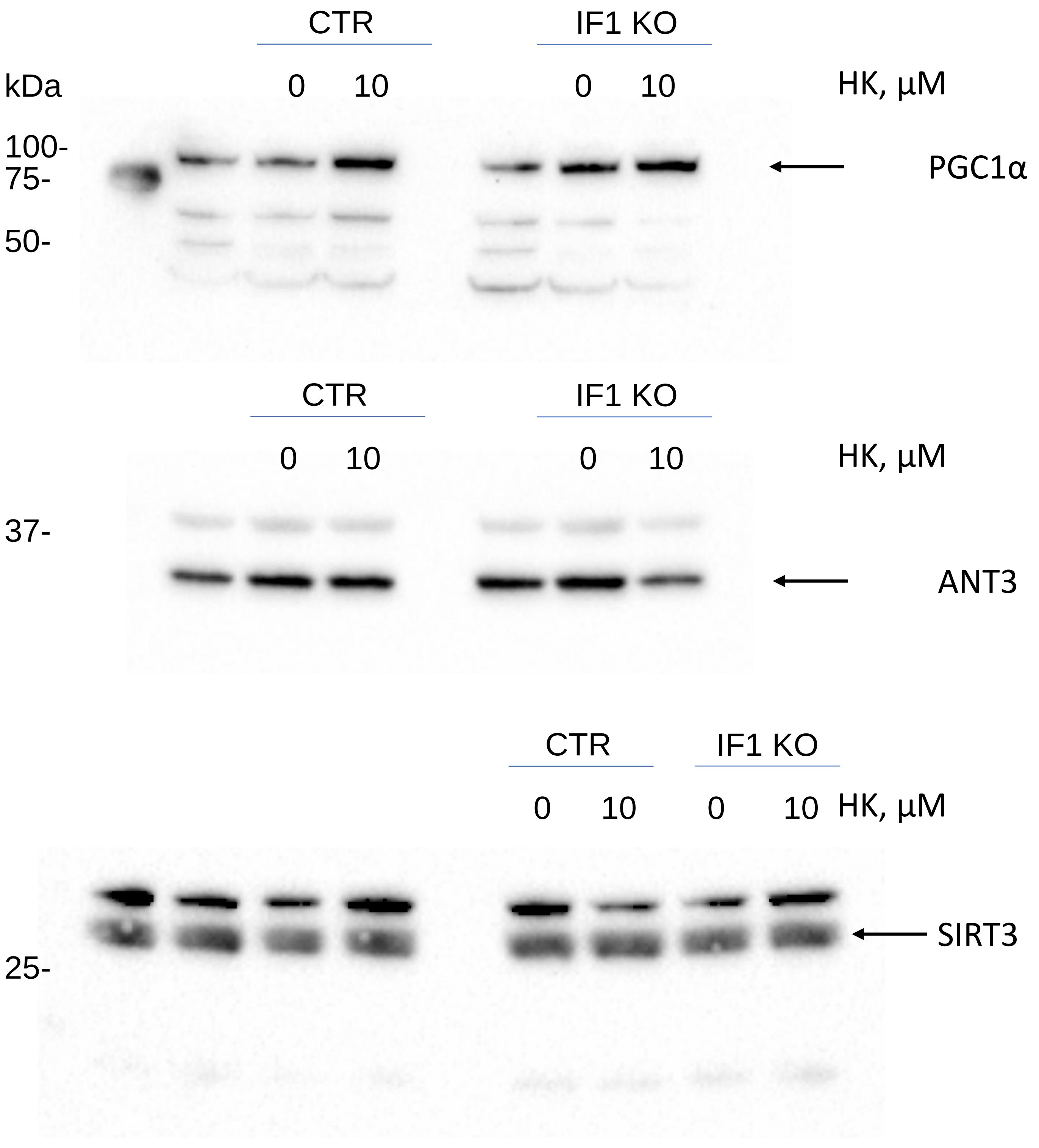

SUPPLEMENTARY FIG. 3B

(i)

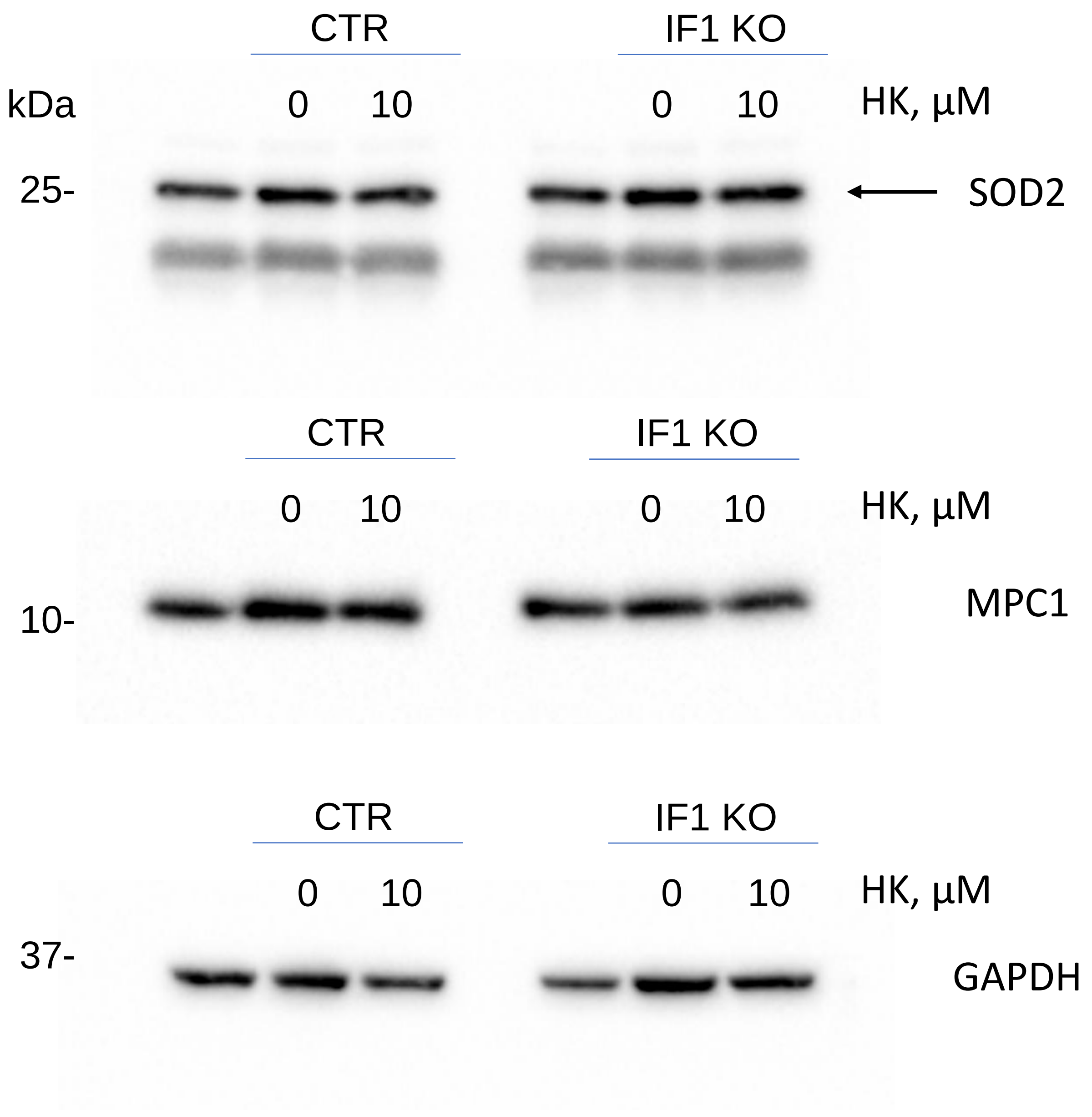

SUPPLEMENTARY FIG. 3C

(i)

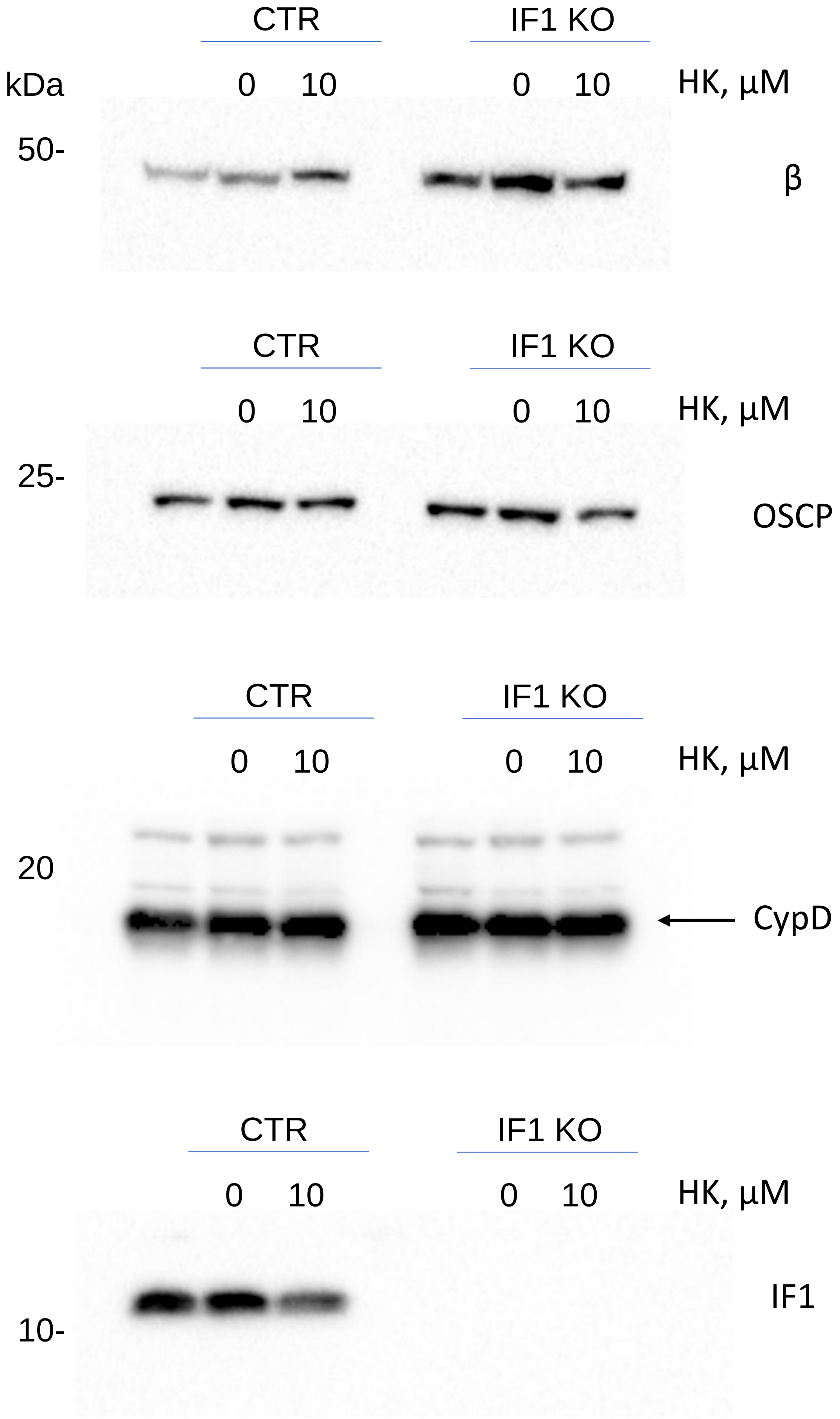

SUPPLEMENTARY FIG. 3C

(i)

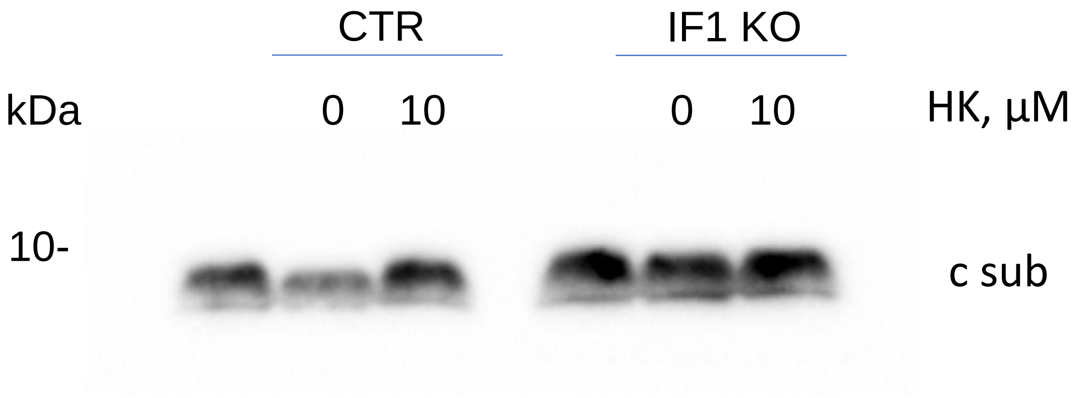

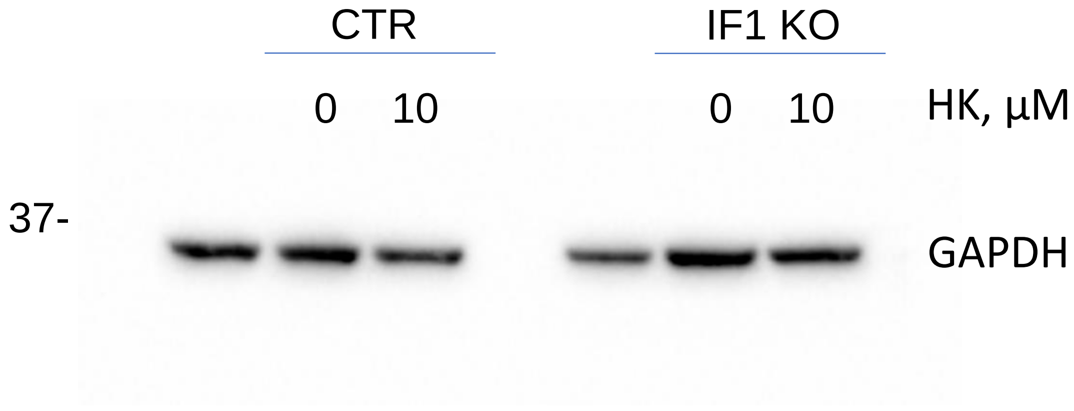

Supplement: Supplementary file 10 — Original blots [file 41419_2026_8441_MOESM10_ESM.pdf]
